# Supplementary material for: Structural basis for dimerization of a paramyxovirus polymerase complex
Source: Nat Commun. 2024 Apr 11;15:3163. doi: 10.1038/s41467-024-47470-7 (PMC11009304; doi:10.1038/s41467-024-47470-7)
Supplement: Supplementary file 1 — Supplementary Information [file 41467_2024_47470_MOESM1_ESM.pdf]

SUPPLEMENTARY INFORMATION

Structural basis for dimerization of a paramyxovirus polymerase complex

Jin Xie[1,6], Mohamed Ouizougun-Oubari[2,6], Li Wang[3,6], Guanglei Zhai[1], Daitze Wu[3], Zhaohu Lin[1], Manfu Wang[4], Barbara Ludeke[2], Xiaodong Yan[4], Tobias Nilsson[5], Lu Gao[3,*], Xinyi Huang[1,*], Rachel Fearns[2,*], Shuai Chen[1,*]

[1]Roche Pharma Research and Early Development, Lead Discovery, Roche Innovation Center Shanghai, Shanghai 201203, China.
[2]Department of Virology, Immunology & Microbiology, Boston University Chobanian & Avedisian School of Medicine, Boston, MA 02118, USA.
[3]Roche Pharma Research and Early Development, Infectious Diseases, Roche Innovation Center Shanghai, Shanghai 201203, China.
[4]Wuxi Biortus Biosciences Co. Ltd., Jiangyin, Jiangsu 214437, China.
[5]Roche Pharma Research and Early Development, Infectious Diseases, Roche Innovation Center Basel, Basel 4070, Switzerland.
[6]These authors contributed equally: Jin Xie, Mohamed Ouizougun-Oubari and Li Wang.
*Corresponding author. Email: goodlucksept2016@yahoo.com (L.G.); xinyi.huang@roche.com (X.H.); rfearns@bu.edu (R.F.); shuai.chen.sc1@roche.com (S.C.).

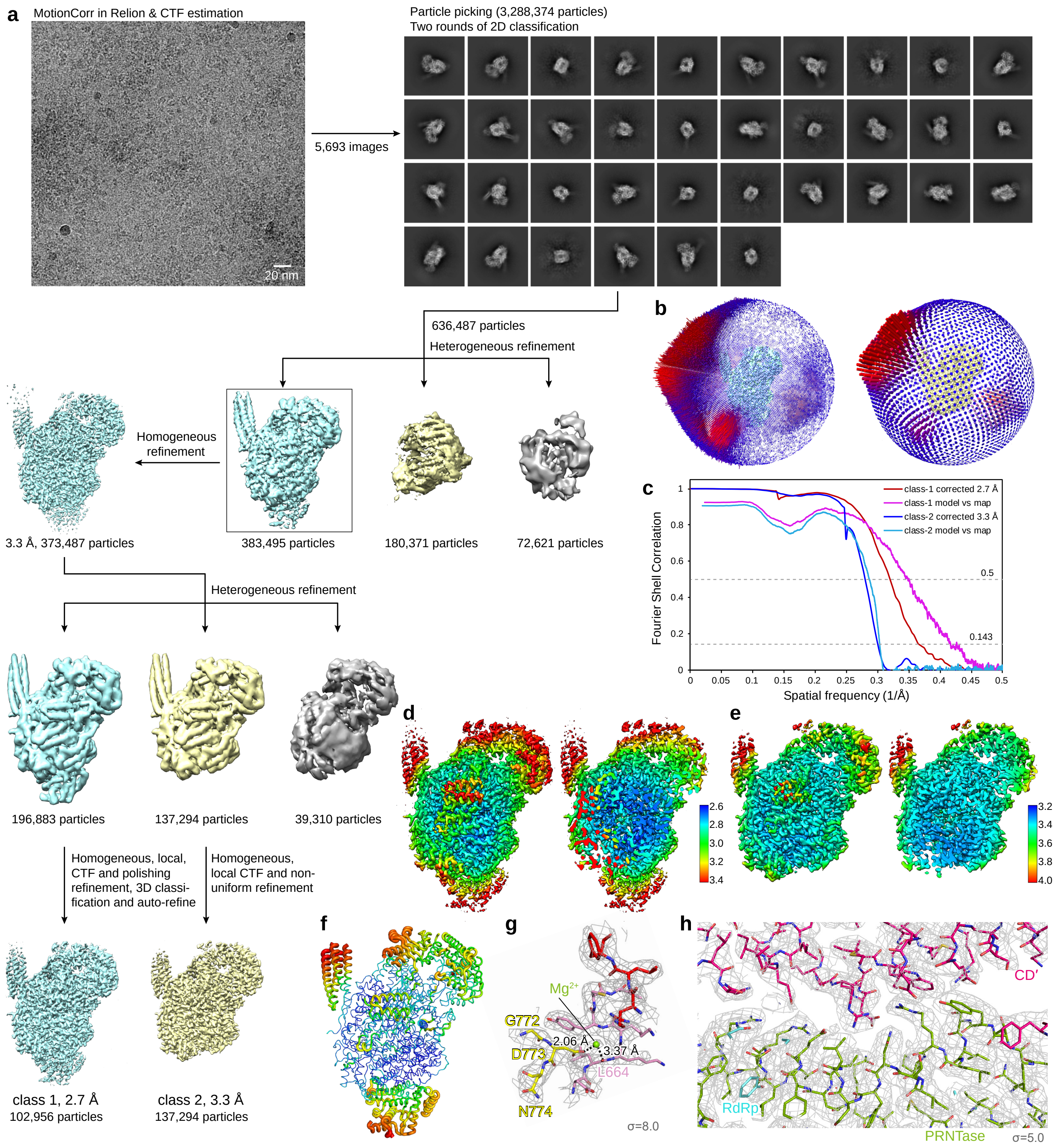

Supplementary Fig. 1. Cryo-EM data processing.

a Flow chart of single particle cryo-EM data processing. **b** Angular distribution of the particles for class 1 (left panel) and class 2 (right panel). **c** The Fourier shell correlation (FSC) curves were used to calculate the resolutions of the final reconstructions with the cutoff of 0.143 and the resolutions are consistent with the model-to-map correlation (0.5 criterion). **d**, **e** Local resolution estimation of the reconstructed cryo-EM density maps of class 1 with CD′ (**d**) and class 2 without CD′ (**e**). Overview on the left and cut-out view on

the right. **f** B-factor analysis of the class 1 structure showing the rigidity and flexibility. The coloring-thickness scale varies from blue and thin (rigid residues) to red and thick (flexible residues). **g**, **h** Representative electron density maps around the RdRp active site including the presumed magnesium ion (Mg^{2+}) and catalytic residues 772-GDN-774 (**g**), and at the L-L dimeric interface between CD′ and RdRp-PRNTase of the intact L (**h**). The maps are shown as grey mesh, contoured at 8.0 and 5.0 σ, respectively. The distances (angstrom, Å) between Mg^{2+} and the oxygen atoms of two coordinated residues are labeled.

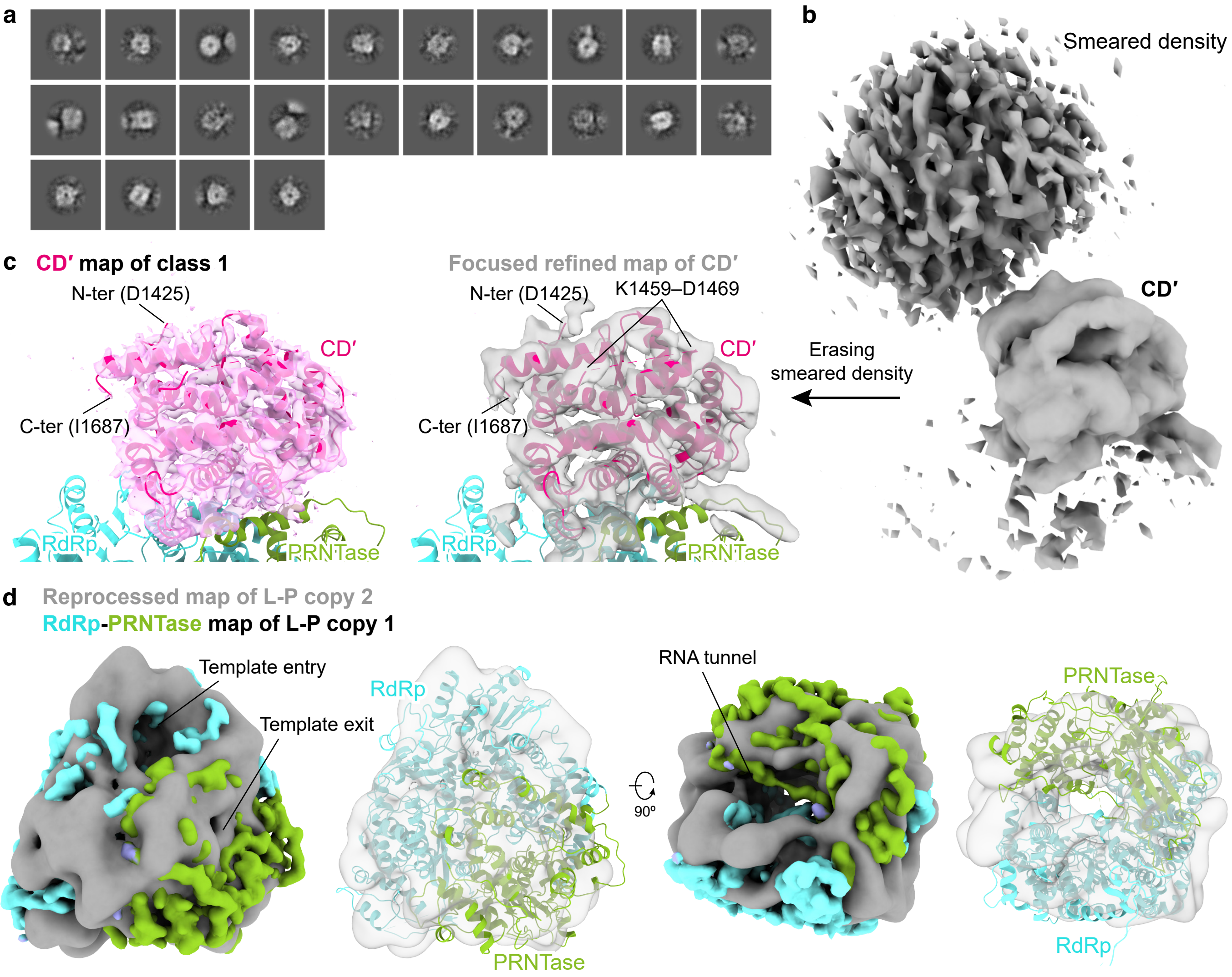

Supplementary Fig. 2. Reconstruction of L-P copy 2 in class 1 particles by subtraction of the "complete" L-P copy.

a 2D classification of L-P copy 2 after subtraction of the "complete" L-P copy 1 from class 1 particles. **b** 3D classification with good CD′ alignments derived from the refinement of class 1. **c** Focused refinement after erasing the smeared density in **b** obtained a 4.5 Å map of CD′. The original CD′ map of class 1 is shown in the left panel. The CD′, RdRp, PRNTase domains are shown in ribbons. The N- and C-terminal residues of CD′ and the visible loop between Lys1459 and Asp1469 of CD′ in the focused refined map are labeled. **d** Reconstruction of the particles from the 2D classes in **a** with re-calculated alignments obtained a map of potential RdRp-PRNTase of L-P copy 2 at 7.0 Å. The RdRp-PRNTase map and structure of L-P copy 1 in class 1 are fitted into the reprocessed map. Rotated view by 90° about the horizontal axis is shown in the right panel. The proposed template entry, template exit and RNA tunnel are labeled.

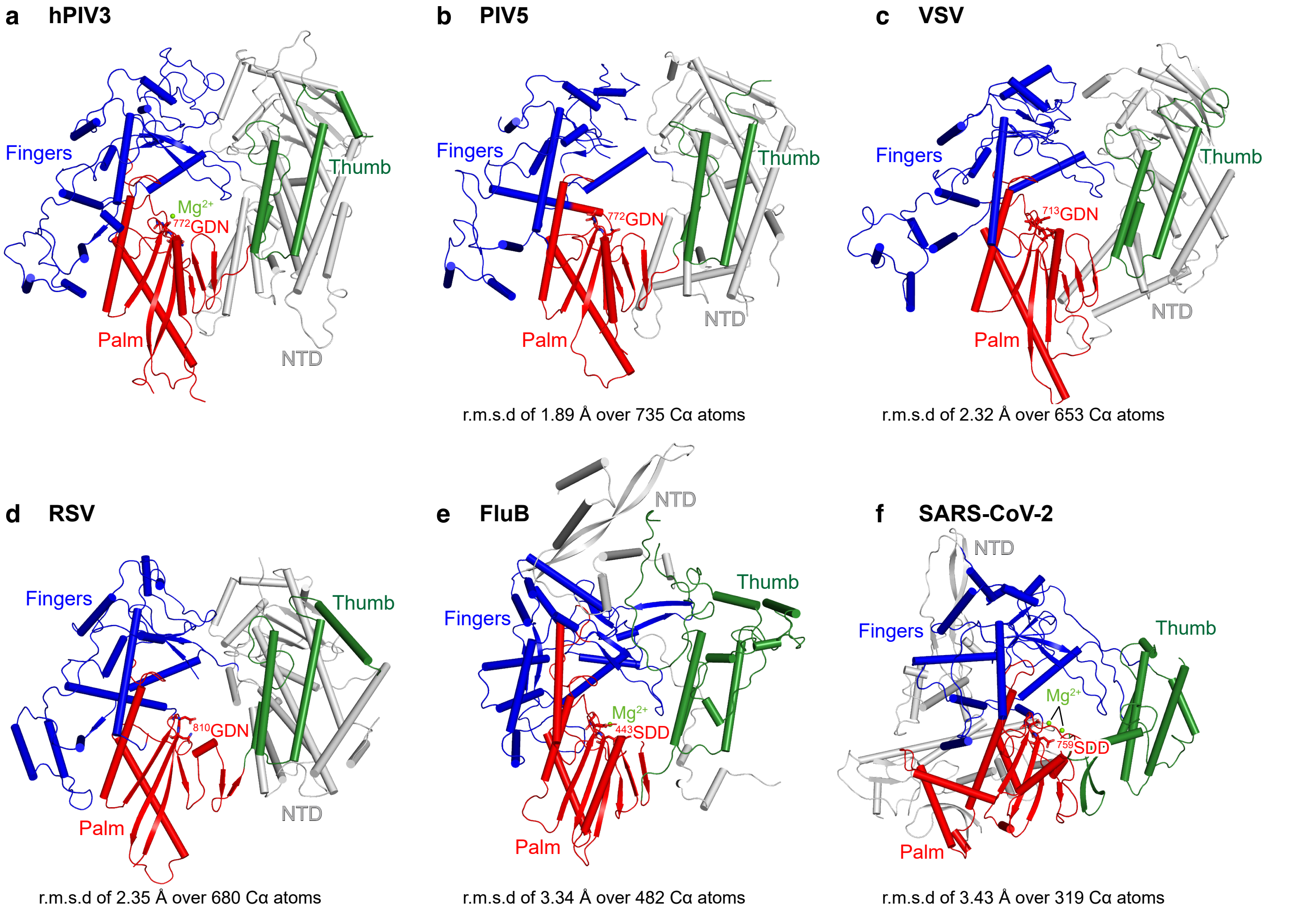

Supplementary Fig. 3. Similarity of the RdRp domains between hPIV3 and other viral polymerases.

The polymerase RdRp domains of hPIV3 (**a**); PIV5 (PDB 6V85) (**b**); VSV (PDB 6U1X) (**c**); RSV (PDB 6PZK) (**d**); FluB (PDB 6QCX) (**e**); SARS-CoV-2 (PDB 7BV2) (**f**). The RdRp subdomains are colored as depicted in Fig. 2a. The catalytic residues and magnesium ions at the active site are shown as sticks and sphere, respectively. Structures are superimposed to hPIV3 RdRp domain including the N-terminal region (NTD) with low structural similarity, and the root mean square deviation (r.m.s.d) value and residue coverage are marked.

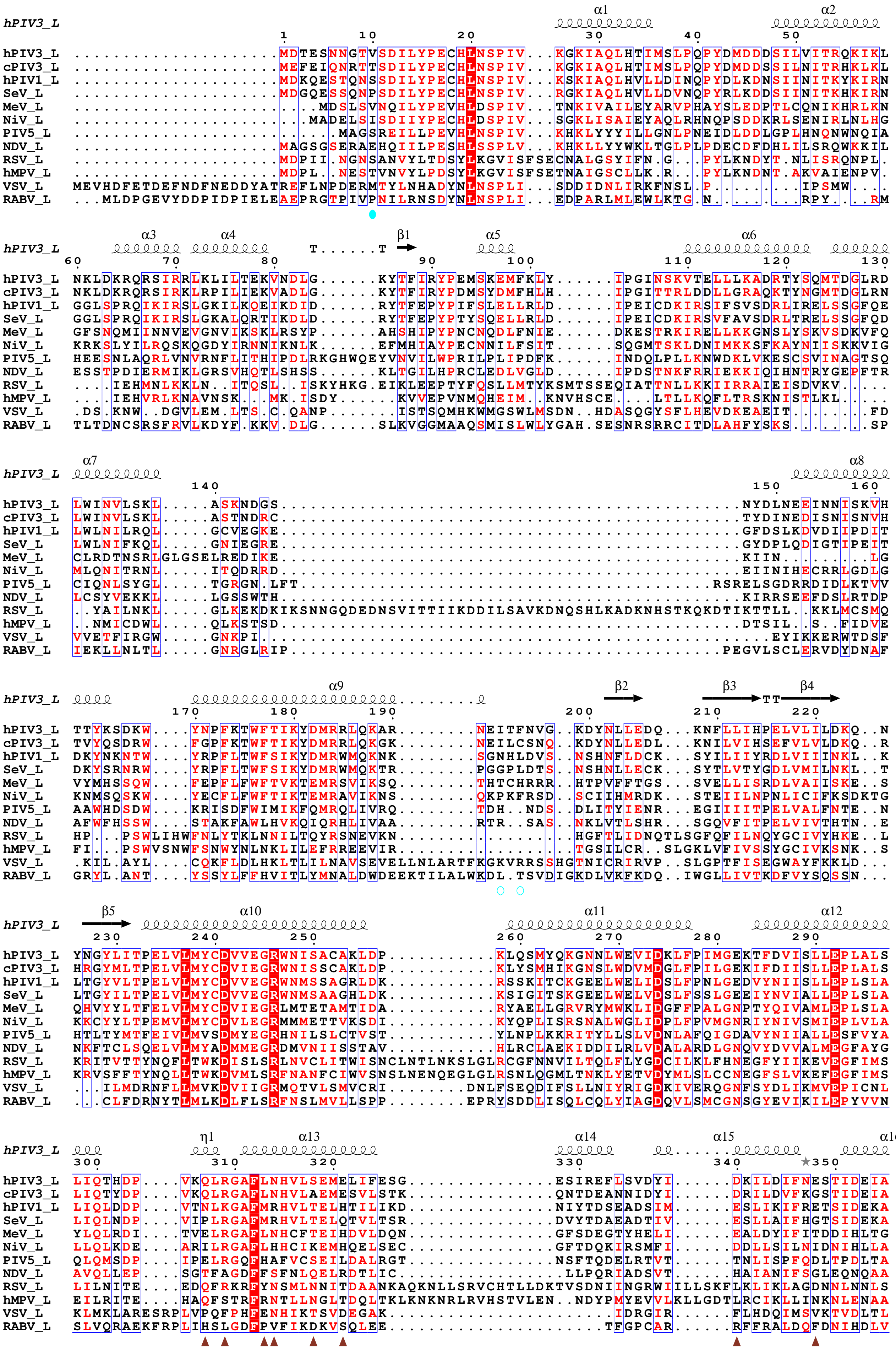

hPIV3_L
α1 α2
1 10 20 30 40 50
hPIV3_L .....................MDTESNNGTVSDILYPECHLNSPIV...KGKIAQLHTIMSLPQPYDMDDDSILVITRQKIKL
cPIV3_L .....................MEFEIQNRTTSDILYPECHLNSPIV...KGKIAQLHTIMSLPQPYDMDDSSILNITRHKLKL
hPIV1_L .....................MDKQESTQNSSDILYPECHLNSPIV...KSKIAQLHVLLDINQPYDLKDNSIINITKYKIRN
SeV_L .....................MDGQESSQNPSDILYPECHLNSPIV...RGKIAQLHVLLDVNQPYRLKDDSIINITKHKIRN
MeV_L .........................MDSLSVNQILYPEVHLDSPIV...TNKIVAILEYARVPHAYSLEDPTLCQNIKHRLKN
NiV_L ........................MADELSISDIIYPECHLDSPIV...SGKLISAIEYAQLRHNQPSDDKRLSENIRLNLHG
PIV5_L ...........................MAGSREILLPEVHLNSPIV...KHKLYYYILLGNLPNEIDLDDLGPLHNQNWNQIA
NDV_L .....................MAGSGSERAEHQIILPESHLSSPLV...KHKLLYYWKLTGLPLPDECDFDHLILSRQWKKIL
RSV_L .....................MDPII.NGNSANVYLTDSYLKGVISFSECNALGSYIFN.G...PYLKNDYT.NLISRQNPL.
hMPV_L .....................MDPL..NESTVNVYLPDSYLKGVISFSETNAIGSCLLK.R...PYLKNDNT.AKVAIENPV.
VSV_L MEVHDFETDEFNDFNEDDYATREFLNPDERMTYLNHADYNLNSPLI...SDDIDNLIRKFNSL.P.........IPSMW....
RABV_L ...MLDPGEVYDDPIDPIELEAEPRGTPIVPNILRNSDYNLNSPLI...EDPARLMLEWLKTG.N.........RPY...RM
hPIV3_L
α3 α4 T......T β1 α5 α6
60 70 80 90 100 110 120 130
hPIV3_L NKLDKRQRSIRRLKLILTEKVNDLG.....KYTFIRYPEMSKEMFKLY......IPGINSKVTELLLKADRTYSQMTDGLRD
cPIV3_L NKLDKRQRSIRKLRPILIEKVADLG.....KYTFIRYPDMSYDMFHLH......IPGITTRLDDLLGRAQKTYNGMTDGLRN
hPIV1_L GGLSPRQIKIRSLGKILKQEIKDID.....RYTFEPYPIFSLELLRLD......IPEICDKIRSIFSVSDRLIRELSSGFQE
SeV_L GGLSPRQIKIRSLGKALQRTIKDLD.....RYTFEPYPTYSQELLRLD......IPEICDKIRSVFAVSDRLTRELSSGFQD
MeV_L GFSNQMIINNVEVGNVIKSKLRSYP.....AHSHIPYPNCNQDLFNIE......DKESTRKIRELLKKGNSLYSKVSDKVFQ
NiV_L KRKSLYILRQSKQGDYIRNNIKNLK.....EFMHIAYPECNNILFSIT......SQGMTSKLDNIMKKSFKAYNIISKKVIG
PIV5_L HEESNLAQRLVNVRNFLITHIPDLRKGHWQEYVNVILWPRILPLIPDFK......INDQLPLLKNWDKLVKESCSVINAGTSQ
NDV_L ESSTPDIERMIKLGRSVHQTLSHSS.....KLTGILHPRCLEDLVGLD......IPDSTNKFRRIEKKIQIHNTRYGEPFTR
RSV_L ....IEHMNLKKLN..ITQSL..ISKYHKG.EIKLEEPTYFQSLLMTYKSMTSSEQIATTNLLKKIIRRAIEISDVKV.....
hMPV_L ....IEHVRLKNAVNSK...MK.ISDY.....KVVEPVNMQHEIM..KNVHSCE....LTLLKQFLTRSKNISTLKL.....
VSV_L .DS.KNW..DGVLEM.LTS.C.QANP......ISTSQMHKWMGSWLMSDN..HDASQGYSFLHEVDKEAEIT........FD
RABV_L TLTDNCSRSFRVLKDYF.KKV.DLG.....SLKVGGMAAQSMISLWLYGAH.SESNRSRRCITDLAHFYSKS........SP
α7 α8
hPIV3_L
140 150 160
hPIV3_L LWINVLSKL.....ASKNDGS..NYDLNEEINNISKVH
cPIV3_L LWINVLSKL.....ASTNDRC..TYDINEDISNISNVH
hPIV1_L LWLNILRQL.....GCVEGKE..GFDSLKDVDIIPDIT
SeV_L LWLNIFKQL.....GNIEGRE..GYDPLQDIGTIPEIT
MeV_L CLRDTNSRLGLGSELREDIKE..KIIN........LG
NiV_L MLQNITRNL.....ITQDRRD..EIINIHECRRLGDLG
PIV5_L CIQNLSYGL.....TGRGN.LFT.......................................RSRELSGDRRDIDLKTVV
NDV_L LCSYVEKKL.....LGSSWTH..KIRRSEEFDSLRTDP
RSV_L ..YAILNKL.....GLKEKDKIKSNNGQDEDNSVITTIIKDDILSAVKDNQSHLKADKNHSTKQKDTIKTTLL..KKLMCSMQ
hMPV_L ..NMICDWL.....QLKSTSD..DTSIL..S..FIDVE
VSV_L VVETFIRGW.....GNKPI...EYIKKERWTDSF
RABV_L IEKLLNLTL.....GNRGLRIP..PEGVLSCLERVDYDNAF
hPIV3_L
α9 β2 β3 TT β4
170 180 190 200 210 220
hPIV3_L TTYKSDKW....YNPFKTWFTIKYDMRRLQKAR........NEITFNVG..KDYNLLEDQ...KNFLLIHPELVLILDKQ..N
cPIV3_L TVYQSDRW....FGPFKTWFTIKYDMRRLQKGK........NEILCSNQ..KDYNLLEDL...KNILVIHSEFVLVLDKQ..R
hPIV1_L DKYNKNTW....YRPFLTWFSIKYDMRWMQKNK........SGNHLDVS..NSHNFLDCK...SYILIIYRDLVIIINKL..K
SeV_L DKYSRNRW....YRPFLTWFSIKYDMRWMQKTR........PGGPLDTS..NSHNLLECK...SYTLVTYGDLVMILNKL..T
MeV_L VYMHSSQW....FEPFLFWFTVKTEMRSVIKSQ........THTCHRRR..HTPVFFTGS...SVELLISRDLVAIISKE..S
NiV_L KNMSQSKW....YECFLFWFTIKTEMRAVIKNS........QKPKFRSD..SCIIHMRDK...STEIILNPNLICIFKSDKTG
PIV5_L AAWHDSDW....KRISDFWIMIKFQMRQLIVRQ........TDH..NDS..DLITYIENR...EGIIIITPELVALFNTE..N
NDV_L AFWFHSSW....STAKFAWLHVKQIQRHLIVAA........RTR..SAS..NKLVTLSHR...SGQVFITPELVIVTHTN..E
RSV_L HP...PSWLIHWFNLYTKLNNILTQYRSNEVKN.....................HGFTLIDNQTLSGFQFILNQYGCIVYHKE..L
hMPV_L FI...PSWVSNWFSNWYNLNKLILEFRREEVIR.....................TGSILCR..SLGKLVFIVSSYGCIVKSNK..S
VSV_L .KIL.AYL....CQKFLDLHKLTLILNAVSEVELLNLARTFKGKVRRSSHGTNICRIRVP..SLGPTFISEGWAYFKKLD...
RABV_L GRYL.ANT....YSSYLFFHVITLYMNALDWDEEKTILALWKDL.TSVDIGKDLVKFKDQ..IWGLLIVTKDFVYSQSSN...
hPIV3_L
β5 α10 α11 α12
230 240 250 260 270 280 290
hPIV3_L YNGYLITPELVLMYCDVVEGRWNISACAKLDP...........KLQSMYQKGNNLWEVIDKLFPIMGEKTFDVISLLEPLALS
cPIV3_L HRGYMLTPELVLMYCDVIEGRWNISSCAKLDP...........KLYSMHIKGNSLWDVMDGLFPILGEKIFDIISLLEPLALS
hPIV1_L LTGYVLTPELVLMYCDVVEGRWNMSSAGRLDK...........RSSKITCKGEELWELIDSLFPNLGEDVYNIISLLEPLSLA
SeV_L LTGYILTPELVLMYCDVVEGRWNMSAAGHLDK...........KSIGITSKGEELWELVDSLFSSLGEEIYNVIALLEPLSLA
MeV_L QHVYYLTFELVLMYCDVIEGRLMTETAMTIDA...........RYAELLGRVRYMWKLIDGFFPALGNPTYQIVAMLEPLSLA
NiV_L KKCYYLTPEMVLMYCDVLEGRMMMETTVKSDI...........KYQPLISRSNALWGLIDPLFPVMGNRIYNIVSMIEPLVLA
PIV5_L HTLTYMTFEIVLMVSDMYEGRHNILSLCTVST...........YLNPLKKRITYLLSLVDNLAFQIGDAVYNIIALLESFVYA
NDV_L NKFTCLSQELVLMYADMMEGRDMVNIISSTAV...........HLRCLAEKIDDILRLVDALARDLGNQVYDVVALMEGFAYG
RSV_L KRITVTTYNQFLTWKDISLSRLNVCLITWISNCLNTLNKSLGLRCGFNNVILTQLFLYGDCILKLFHNEGFYIIKEVEGFIMS
hMPV_L KRVSFFTYNQLLTWKDVMLSRFNANFCIWVSNSLNENQEGLGLRSNLQGMLTNKLYETVDYMLSLCCNEGFSLVKEFEGFIMS
VSV_L ...ILMDRNFLLMVKDVIIGRMQTVLSMVCRI........DNLFSEQDIFSLLNIYRIGDKIVERQGNFSYDLIKMVEPICNL
RABV_L ...CLFDRNYTLMLKDLFLSRFNSLMVLLSPP........EPRYSDDLISQLCQLYIAGDQVLSMCGNSGYEVIKILEPYVVN
hPIV3_L
η1 α13 α14 α15 α16
300 310 320 330 340 350
hPIV3_L LIQTHDP....VKQLRGAFLNHVLSEMELIFESG................ESIREFLSVDYI......DKILDIFNESTIDEIA
cPIV3_L LIQTYDP....VKQLRGAFLNHVLAEMESVLSTK................QNTDEANNIDYI......DRILDVFKGSTIDEIA
hPIV1_L LIQLDDP....VTNLKGAFMRHVLTELHTILIKD................NIYTDSEADSIM......ESLIKIFRETSIDEKA
SeV_L LIQLNDP....VIPLRGAFMRHVLTELQTVLTSR................DVYTDAEADTIV......ESLLAIFHGTSIDEKA
MeV_L YLQLRDI....TVELRGAFLNHCFTEIHDVLDQN................GFSDEGTYHELI......EALDYIFITDDIHLTG
NiV_L LLQLKDE....ARILRGAFLHHCIKEMHQELSEC................GFTDQKIRSMFI......DDLLSILNIDNIHLLA
PIV5_L QLQMSDP....IPELRGQFHAFVCSEILDALRGT................NSFTQDELRTVT......TNLISPFQDLTPDLTA
NDV_L AVQLLEP....SGTFAGDFFSFNLQELRDTLIC.................LLPQRIADSVT......HAIANIFSGLEQNQAA
RSV_L LILNITE....EDQFRKRFYNSMLNNITDAANKAQKNLLSRVCHTLLDKTVSDNIINGRWIILLSKFLKLIKLAGDNNLNNLS
hMPV_L EILRITE....HAQFSTRFRNTLLNGLTDQLTKLKNKNRLRVHSTVLEN..NDYPMYEVVLKLLGDTLRCIKLLINKNLENAA
VSV_L KLMKLARESRPLVPQFPHFENHIKTSVDEGAK..................IDRGIR......FLHDQIMSVKTVDLTL
RABV_L SLVQRAEKFRPLIHSLGDFPVFIKDKVSQLEE.................TFGPCAR......RFFRALDQFDNIHDLV

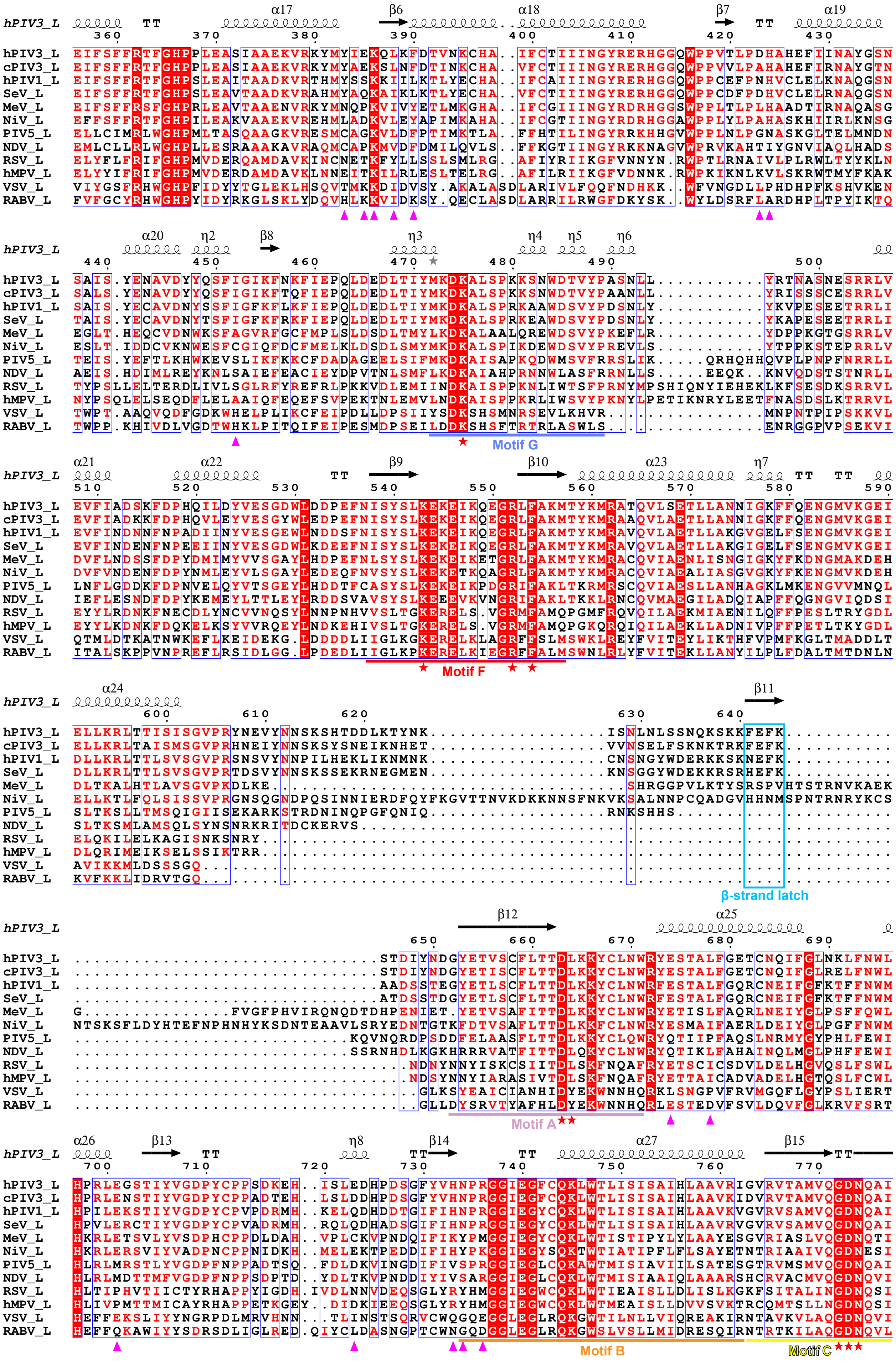
hPIV3_L
α17
β6
α18
β7
α19
α20
η2
β8
η3
η4
η5
η6
α21
α22
β9
β10
α23
η7
α24
β11
β12
α25
α26
β13
η8
β14
α27
β15
hPIV3_L
cPIV3_L
hPIV1_L
SeV_L
MeV_L
NiV_L
PIV5_L
NDV_L
RSV_L
hMPV_L
VSV_L
RABV_L
Motif G
Motif F
β-strand latch
Motif A
Motif B
Motif C

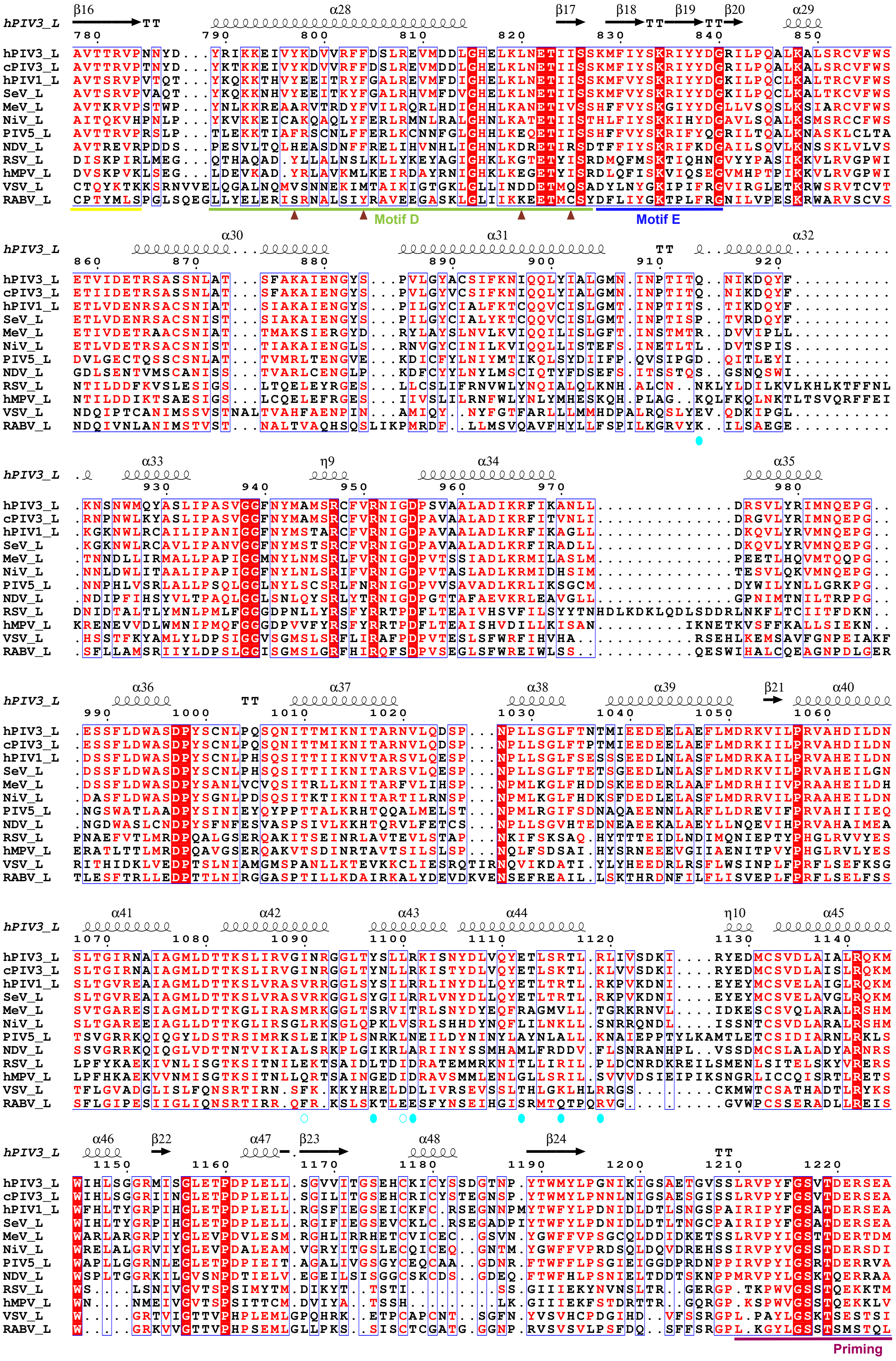

hPIV3_L
cPIV3_L
hPIV1_L
SeV_L
MeV_L
NiV_L
PIV5_L
NDV_L
RSV_L
hMPV_L
VSV_L
RABV_L
Motif D
Motif E
Priming

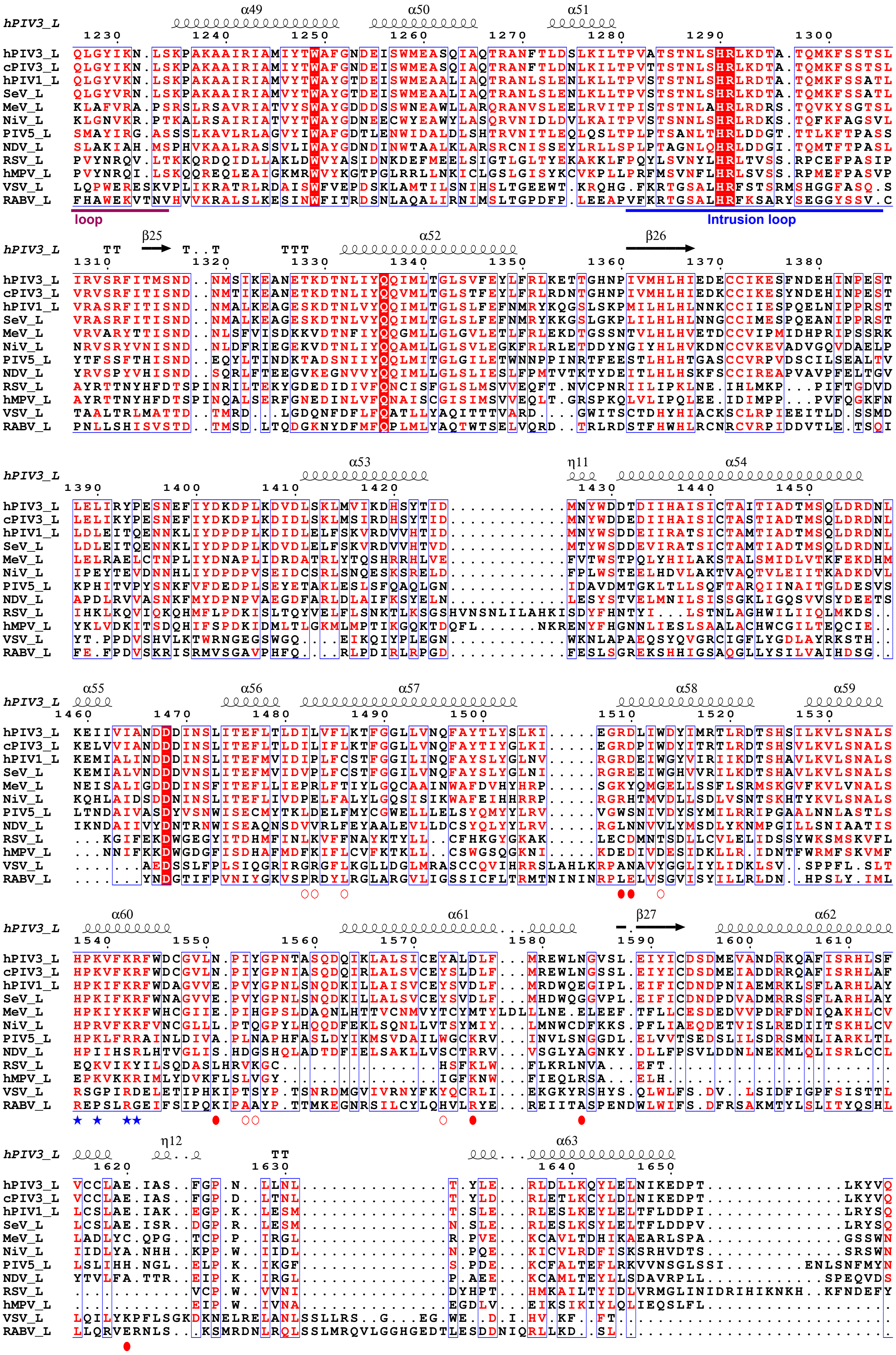
hPIV3_L
cPIV3_L
hPIV1_L
SeV_L
MeV_L
NiV_L
PIV5_L
NDV_L
RSV_L
hMPV_L
VSV_L
RABV_L
loop
Intrusion loop
α49
α50
α51
β25
α52
β26
α53
η11
α54
α55
α56
α57
α58
α59
α60
α61
β27
α62
η12
α63

```
hPIV3_L        β28        α64                                          α65
             1660      1670      1680           1690        1700       1710       1720
hPIV3_L  ISGLLIKSFPSTVTYVRKTAIKYLRIRGISP......PE..VIDDWDPIEDENMLDNIVKT..INDNCN..KDNKGNK.....
cPIV3_L  VSGLLIKSFPSTVTYVRKTAIKHLRIRGISP......PS..IIEDWDPVEDENILDNIAKV..ISDNCDNNWSKNKNK.....
hPIV1_L  LTGLVIKIFPSTLTYIRKSSIKVLRVRGIGI......PE..VLEDWDPDADSMLLDNITAE..VQHNIP....LKKNE.....
SeV_L    LTGLVIKVFPSTLTYIRKSSIKVLRTRGIGV......PE..VLEDWDPEADNALLDGIAAE..IQQNIP....LGHQT.....
MeV_L    INPIIVDHYSCSLTYLRRGSIKQIR....L......RV..D..PGFIFDALAEVNVS.....QPKVGS..............
NiV_L    TSDLDFVIFYASLTYLRRGIIKQLRIRQVTE......VI..D..TTTMLRDNIIVENP....PIKTGV..............
PIV5_L   VENPRLAAFASNNYYLTRKLLNSIRDTESGQ......VA..VTSYYESLEYIDSLKLTPHV..PGTSCI..ED.........
NDV_L    ITSPSIVTFPANLYYMSRKSLNLIREREDRDSI....LALMFPQ....EPLFEFPLVQDIGARVK.................
RSV_L    TSNLFYINY..NFSDNTHLLTKHIRIANSELENNYNKLYHPTPETLENILANPIKSNDKKTLNDYCIGKNVDSIMLPLLSNKK
hMPV_L   ....RITVL..NYTDMAHALTRLIRKKLMCDNA....LLTPISSP....MVNLTQVIDPT.......................
VSV_L    ...KDILLCP.E..EIRHAC.K.FG...IA.......KDNNKDMSYPP...................................
RABV_L   ...RRTR.WVDQ..EVRHAA.RTMTG.DYS.......P.N.KKVSRKVG..................................

hPIV3_L                    TT     β29      TT                                TT    η13
                          1730      1740                  1750      1760      1770
hPIV3_L  ............INNFWGLALKNYQVLKI...RSITSD.........SDDNDRLDASTSGLTLPQGGNYLSHQLR.LFGI
cPIV3_L  ............CNYFWGLSLKNYQVIKV...RSITIN.........SEFPETSIQTNHGLLLPEGGNYLSHQLR.LFGV
hPIV1_L  ............RTPFWGLRVSKSQVLRL...RGYE...........EIKREERGRSGVGLTLPFDGRYLSHQLR.LFGI
SeV_L    ............RAPFWGLRVSKSQVLRL...RGYK...........EITRGEIGRSGVGLTLPFDGRYLSHQLR.LFGI
MeV_L    .................NNISNMSIKDFRPPHDDVAKLL........KDINTSKHNLPI...SGGSLANYEIHAFR.RIGL
NiV_L    ..................LDIRGCIIYNL...EEIL............SMNTKSASKKI..FNLNSRPSVENHKYR.RIGL
PIV5_L   ..............DSLCT.NDYIIWII...ESNANLEK..YP.....IPNSPED.DSN..FHNFKLNAPSHHTLR.PLGL
NDV_L    ..............DQLTMKPAAFLHELD...LSAPAR...YDAY...TLEQARS.DCA..LADMGEDQLVRYLFR.GVGT
RSV_L    LIKSSAMIRTNYSKQDLYN.LFPMVVIDRI...IDHSGNTAKSNQLYTTTSHQISLVHNS..TSLY.CMLPWHHINRFNFVF
hMPV_L   .............TQL.DYFPKITFERL...KNYD...........TSSNYAKGKLT..RNYM.ILLPWQHVNRYNFVF
VSV_L    ............WGRESRGT..ITTIPV...YYTTTPY.PKM.....LEMP...........PRIQNPLLSGIR.LGQL
RABV_L   ............CSEWVCSA..QQV.AV...STSAN..PAPVS.ELDIRALSK...............RFQNPLISGLR.VVQW

hPIV3_L           α66              β30         α67            β31    η14   TT         α68
        1780      1790      1800          1810      1820          1830      1840          1850
hPIV3_L  NSTSCLKALELSQILMKEVNKD..KDRLFLGEGAGAMLACYDAT.LGPAINYYNSGLNITDVIGQRELKI.FPSEVSLV..GK
cPIV3_L  NSTSCLKALELSQILMREVSKD..KDRLFLGEGAGAMLACYDAT.LGPAMNYYNSGLNITDVVGQRELKI.FPSEVSLV..GK
hPIV1_L  NSTSCLKALELTYLLNPLVNKD..KDRLYLGEGAGAMLSCYDAT.LGPCMNYYNSGVNSCDLNGQRELNI.YPSEVALV..GK
SeV_L    NSTSCLKALELTYLLSPLVDKD..KDRLYLGEGAGAMLSCYDAT.LGPCINYYNSGVYSCDVNGQRELNI.YPAEVALV..GK
MeV_L    NSSACYKAVEISTLIRRCLEPG..EDGLFLGEGSGSMLITYKEI.LKLNKCFYNSGVSANSRSGQRELAP.YPSEVGLV..EH
NiV_L    NSSSCYKALNLSPLIQRYLPSG..AQRLFIGEGSGSMMLLYQST.LGQSISFYNSGIDGDYIPGQRELKL.FPSEYSIA..EE
PIV5_L   SSTAWYKGISCCRYLERLKLPQ..GDHLYIAEGSGASMTIIEYL.FPGRKIYYNSLFSSGDNPPQRNYAP.MPTQFIESVPYK
NDV_L    ASSSWYKASHLLSVPEIRCARH..GNSLYLAEGSGAIMSLLELH.IPHETIYYNTLFSNEMNPPQRHFGP.TPTQFLNSVVYR
RSV_L    SSTGCK..ISIEY.ILKDLKIKDPNCIAFIGEGAGNLLLRTVVELHPDIRYIYRSLKDCNDHS........LPIEFLRL..YN
hMPV_L   SSTGCK..VSLKTCIGKLMKDLNPKVLYFIGEGAGNWMARTACE.YPDIKFVYRSLKDDLDHH........YPLEYQRV..IG
VSV_L    PTGAHYK...IRSILHGMGIHY..RDFLSCGDGSGGMTAALLRE.NVHSRGIFNSLLELSGSVM.RGASPEPPSALETL..GG
RABV_L   ATGAHYK...LKPILDDLNVFP..SLCLVVGDGSGGISRAVLNM.FPDAKLVFNSLLEVNDLMA.SGTHPLPPSAIMRG..GN

hPIV3_L               η15  β32         η16       α69              β33                α70
               1860          1870          1880      1890          1900      1910      1920      1930
hPIV3_L  K....LGNVTQILNRVKVLF.NGNPNSTWIGNMECESLIWSELN..DKSIGLVHCDMEGAIGKSEETVLHEHYSVIRITYLI.
cPIV3_L  K....LGNVTQILNRVKVLF.NGNPNSTWIGNMECESLIWSELN..DKSIGLVHCDMEGAIGKSEETVLHEHYSVIRITYLV.
hPIV1_L  K....LNNVTSLCQRVKVLF.NGNPGSTWIGNDECEALIWNELQ..NNSIGFIHCDMEGGEHKCDQVVLHEHYSVIRIAYLV.
SeV_L    K....LNNVTSLGQRVKVLF.NGNPGSTWIGNDECEALIWNELQ..NSSIGLVHCDMEGGDHKDDQVVLHEHYSVIRIAYLV.
MeV_L    R........MGVGNIVKVLF.NGRPEVTWVGSIDCFNFIVSNIP..TSSVGFIHSDIETLPNKDTIEKLEELAAILSMALLL.
NiV_L    D....PSLTGKLKGLVVPLF.NGRPETTWIGNLDSYEYIINRTA..GRSIGLVHSDMESGIDKNVEEILVEHSHLISIAINV.
PIV5_L   LWQAHTDQYPEIFEDFIPLW.NGNAAMTDIGMTACVEFIINRVG..PRTCSLVHVDLESSASLNQQCLSKPIINAIITATTV.
NDV_L    NLQAEVPCKDGFVQEFRTLW.RENTEESDLTSDKAVGYITSVVP..YRSVSLLHCDIEIPPGSNQSLLDQLATNLSLIAMHS.
RSV_L    G..........HINIDYGENLTIPATDATNNIHWSYLHIKF...AEPISLFVCDAELSV...TVNWSKIIIEWSKHVRKCK
hMPV_L   E..........LSRIIDSGEGLSMETTDATQKTHWDLIHRVS...KDALLITLCDAEFKD...RDDFFKMVILWRKHVLSCR
VSV_L    D..........KSRCVNGE.TCWEYPSDLCDPRTWDYFLRLKAGLGLQIDLIVMDMEVR...DSSTSLKIETNVRNYVHRI.
RABV_L   D..........IVSRVIDLD.SIWEKPSDLRNLATWKYFQSVQKQVNMSYDLIICDAEVT...DIASINRITLL.MSDFALS.

hPIV3_L        β34                     α71       β35         TT     β36              α72
                   1940          1950      1960      1970      1980      1990      2000
hPIV3_L  ..GDD..DVVLVSKIIPT.ITPNWSRILYLYKLYWKDVSIISLKTSNPASTELYLISKDAYCTIMEPSEVVLSKLKRLS....
cPIV3_L  ..GDD..DVILVSKIIPS.LTPNWSKIITLYKLYWKDVRIISLKTSNPASTELYLISKEAYCTVMEPSSVILSKLNRIS....
hPIV1_L  ..GDK..DVILVSKIAPR.LGTDWTKQLSLYLRYWRDVSLIVLKTSNPASTEMYLISKDPKSDIIEDSNTVLANLLPLS....
SeV_L    ..GDR..DVVLISKIAPR.LGTDWTRQLSLYLRYWDEVNLIVLKTSNPASTEMYLLSRHPKSDIIEDSKTVLASLLPLS....
MeV_L    ..GKI..GSILVIKLMPF.SGDFVQGFISYVGSHYREVNLVYPRYSNFISTESYLVMTDLKANRLMNPEKIKQQIIESS....
NiV_L    ..MME..DGLLVSKIAYT.PGFPISRLFNMYRSYFGLVLVCFPVYSNPDSTEVYLLCLQKTVKTIVPPQKVLEHSNLHD....
PIV5_L   ..LCP..HGVLILKYSWL.PFTRFSTLITFLWCYFERITVLRSTYSDPANHEVYLICILANNFAFQTVSQATGMAMTLTDQGF
NDV_L    ..VRE..GGVVIVKILYS.MGYYFHLLVNLFTPCSVKGYVLSNGYACRGDMECYVVFVMGYLGGPTFVNEVVRMAKTLIQRHG
RSV_L    YCSSV.NKCMLIVKYHA.......QDDIDFKLDNITILKTYVCLGSKLKGSEVYLVLTIGPANIFP.................
hMPV_L   ICTTYGTDLYLFAKYHA......KDCNVKLPFFVRSVATFIMQGSKLSGSECYILLTLGHHNSLP..................
VSV_L    ..LDE..QGVLIYKTYGTYICESEKNAVTILGPMFKTVDLVQTEFSSSQTSEVYMVCKGLKKLIDE......P.N.PD.....
RABV_L   ..IDG..PLYLVFKTYGTMLVNPNYKAIQHLSRAFPSVTGFITQVTSSFSSELYLRFSKRGKF.FRDA.....E.Y.LT....

hPIV3_L                    α73                                    α74           α75
          2010      2020                2030          2040      2050         2060
hPIV3_L  LLEENNLLKWIILSKKRNNEWL.......HHEIKEG.ER..DYGVMRPYHMALQIFG..FQINLNHLAKEFLSTPD.......
cPIV3_L  PMEENNLLKWMIISKKKNSEWL.......RHEINEG.ER..DYGIMRPYHTALQIFG..FQINLNHLAREFLTSPD.......
hPIV1_L  KEDSIKIEKWILVEKAKVHDWI.......VRELKEG.SA..SSGMLRPYHQALQIFG..FEPNLNKLCRDFLSTLN.......
SeV_L    KEDSIKIEKWILIEKAKAHEWV.......TRELREG.SS..SSGMLRPYHQALQTFG..FEPNLYKLSRDFLSTMN.......
MeV_L    VRTSPGLIGHILSIKQLSCIQAIVGGAVSRGDINP..IL..KK..LTPIEQVLISCG..LAINGPKLCKELI.........
NiV_L    EVNDQGITSVIFKIKNSQSKQFHDDLKKYYQIDQPFFVP..TK..ITSDEQVLLQAG..LKLNGPEILKSEI.........
PIV5_L   TLISPERINQYWDGHLKQERIV.......AEAIDKV.VL..GENALFNSSDNELILKCGGTPNARNLIDIEP.........
NDV_L    TLLAKSDETALMALFTSQKQRV.......DNILSSP.LPRLAKLLRRNIDTALIEAG..GQPVRPFCAESLVNT........
RSV_L    .....VFNVVQNAKLILSRTK.......NFIMPKKADKE..................SIDANIKSLIPFLCYPI.........
hMPV_L   .....CHGEIQNSKMKIAVCN.......DFYAAKKLD....................NKSIEANCKSLLSGLRI.......
VSV_L    WSSINESWKNLYAFQSSEQEFA.......RAKKVS.T........YFTLTGIPSQ..FIPDPFVNIETMLQIFGVPTGVSH
RABV_L   SSTLREMSLVLFNCSSPKSEMQ.......RARSLNY.........QDLVRGFPEE..IISNPYNEMIITLIDSDVESFLVH
```

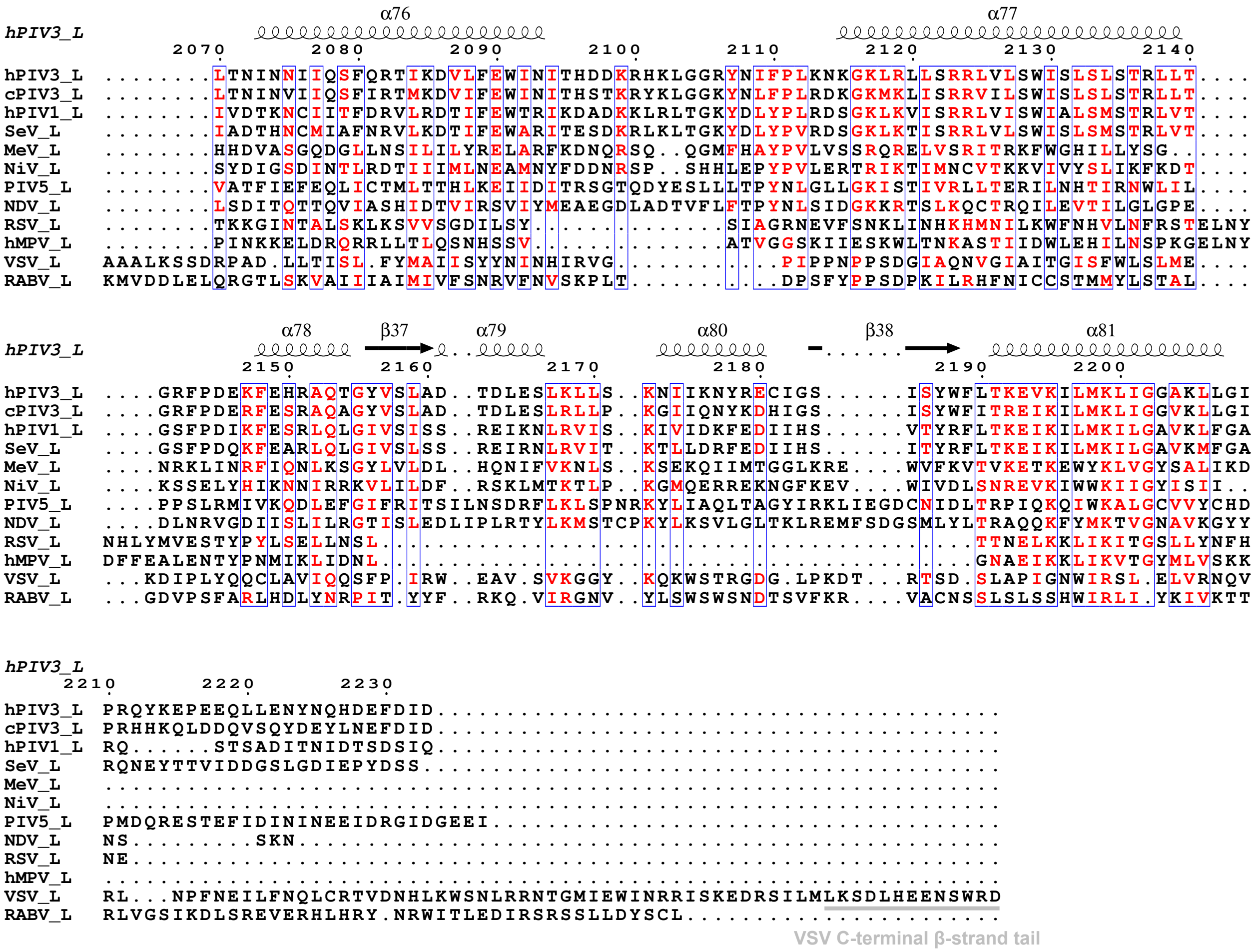

Supplementary Fig. 4. Structure-based sequence alignment of nsNSV L proteins.

Secondary structure elements of hPIV3 L are shown above the alignment. The presumed catalytic residues and critical residues at the RdRp active site are indicated by red stars below the alignment. The residues involved in L-L interactions of hPIV3 L are indicated by circles: residues on the RdRp and PRNTase domains of the intact L and the CD domain (CD′) of the second L are colored in cyan and red, respectively; residues participated in polar and hydrophobic interactions are shown as solid and hollow circles, respectively. The basic residues that constitute the positively charged surface of CD′ connected to the putative template RNA entry of the adjoining L are shown as blue stars. The residues of hPIV3 L involved in the interactions with P-OD and P-XD are indicated by pink and maroon triangles, respectively. Motifs A–G of the RdRp domain, the priming loop and intrusion loop of the PRNTase domain, the β-strand latch of hPIV3 L RdRp and C-terminal β-strand tail of VSV L CTD are labeled. The sequences of L proteins are from the following viruses: hPIV3 (NP_067153.2), caprine parainfluenza virus 3 (cPIV3, YP_009179212.1), human parainfluenza virus 1 (hPIV1, NP_604442.1), Sendai virus (SeV, BAN84672.1), Measles virus (MeV, NP_056924.1), Nipah virus (NiV, NP_112028.1), parainfluenza virus 5 (PIV5, YP_138518.1), Newcastle disease virus (NDV, ALP75897.1), respiratory syncytial virus (RSV, YP_009518860.1), human metapneumovirus (hMPV, Q6WB93), vesicular stomatitis virus (VSV, NP_041716.1) and rabies virus (RABV, ABN11300.1).

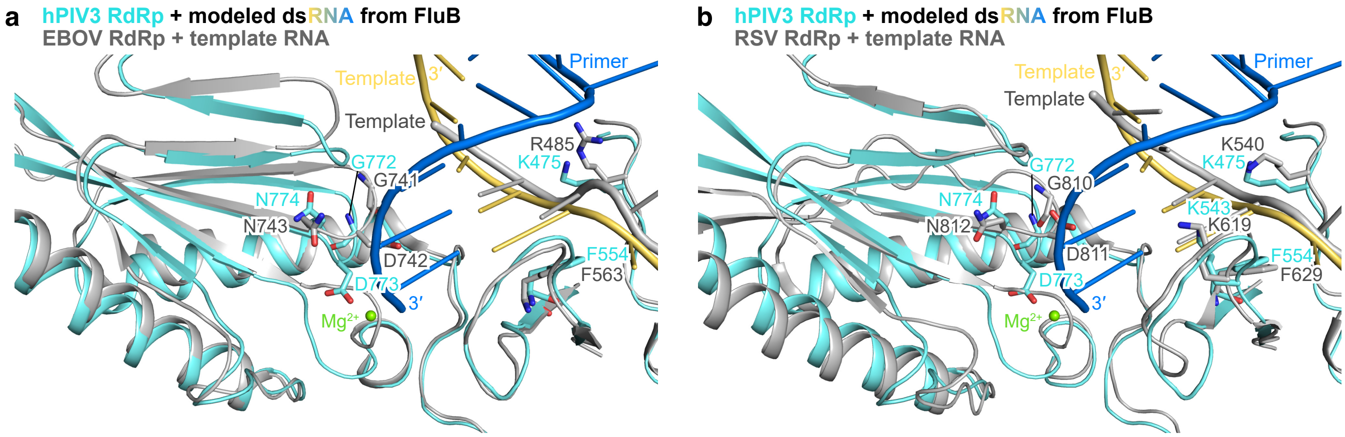

Supplementary Fig. 5. Comparison of hPIV3 RdRp active site with modeled dsRNA to EBOV (a) and RSV (b) RdRp active site bound to the template strand RNA.

The RdRp active site of hPIV3 L is colored in cyan, and the template and primer (product) strand RNAs of the modeled dsRNA from FluB RdRp structure (PDB 6QCX) are colored in light orange and marine blue, respectively. The RdRp active site and the template strand RNA of EBOV (PDB 8JSN) and RSV (PDB 8SNX) structures are colored in grey. Three critical residues identified in hPIV3 structure and the corresponding residues that interact with the 10-nt template strand RNA in EBOV and RSV structures, as well as the catalytic residues are shown as sticks. The magnesium ion in hPIV3 structure is shown as sphere.

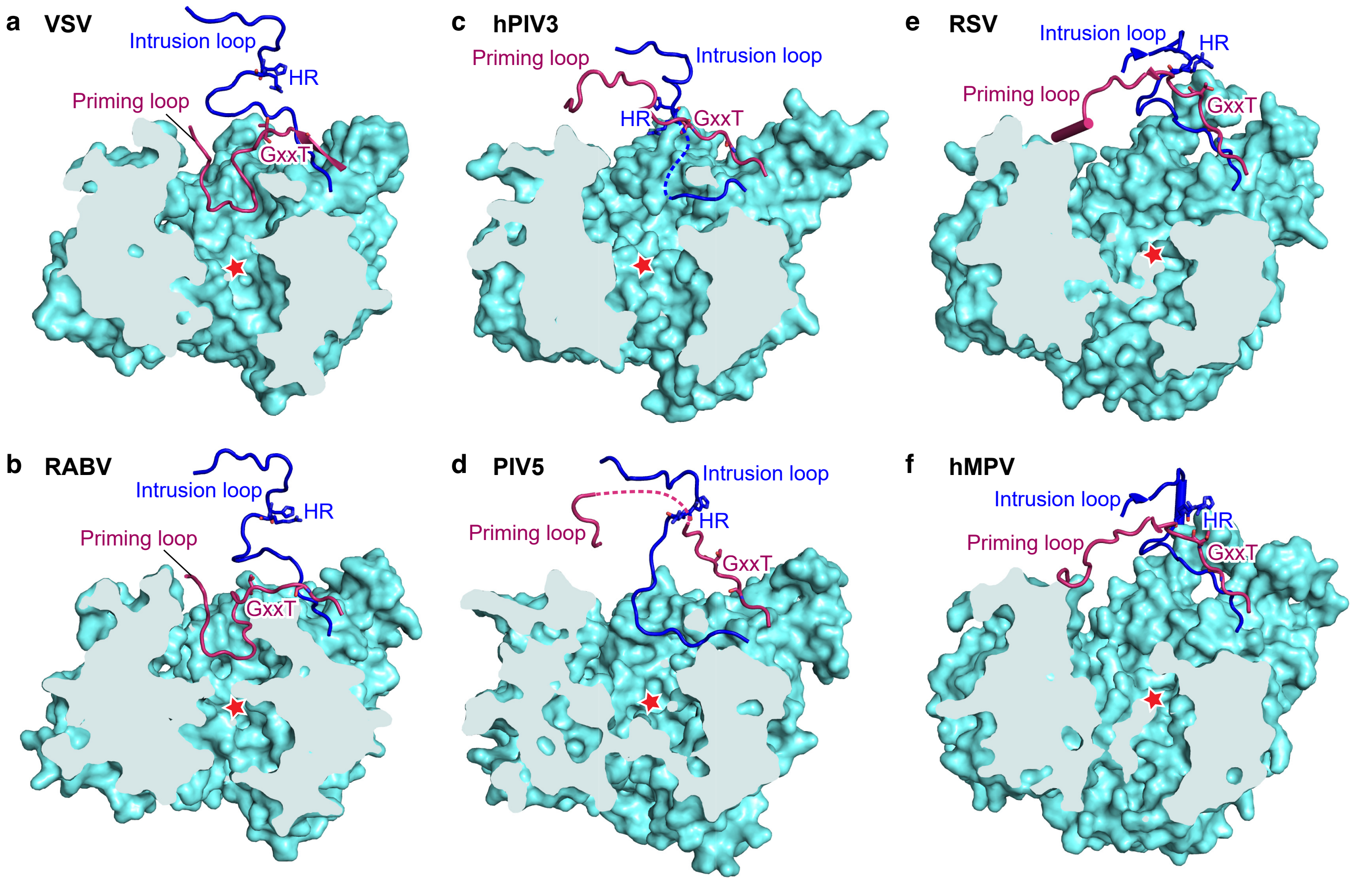

Supplementary Fig. 6. Comparison of the putative priming loops and intrusion loops between hPIV3 and other nsNSV polymerases.

a VSV (PDB 6U1X); **b** RABV (PDB 6UEB); **c** hPIV3; **d** PIV5 (PDB 6V85); **e** RSV (PDB 6PZK); **f** hMPV (PDB 6U5O). The RdRp domains are shown as aquamarine surface at the same view as in Fig. 2e, while the priming and intrusion loops in PRNTase are shown as ribbons in claret and blue, respectively. The conserved GxxT motif and HR motif are shown as sticks. The HR motif is directed towards the RNA cavity in the hPIV3 structure, but situated away from the cavity in other structures. The red star indicates the RdRp active site.

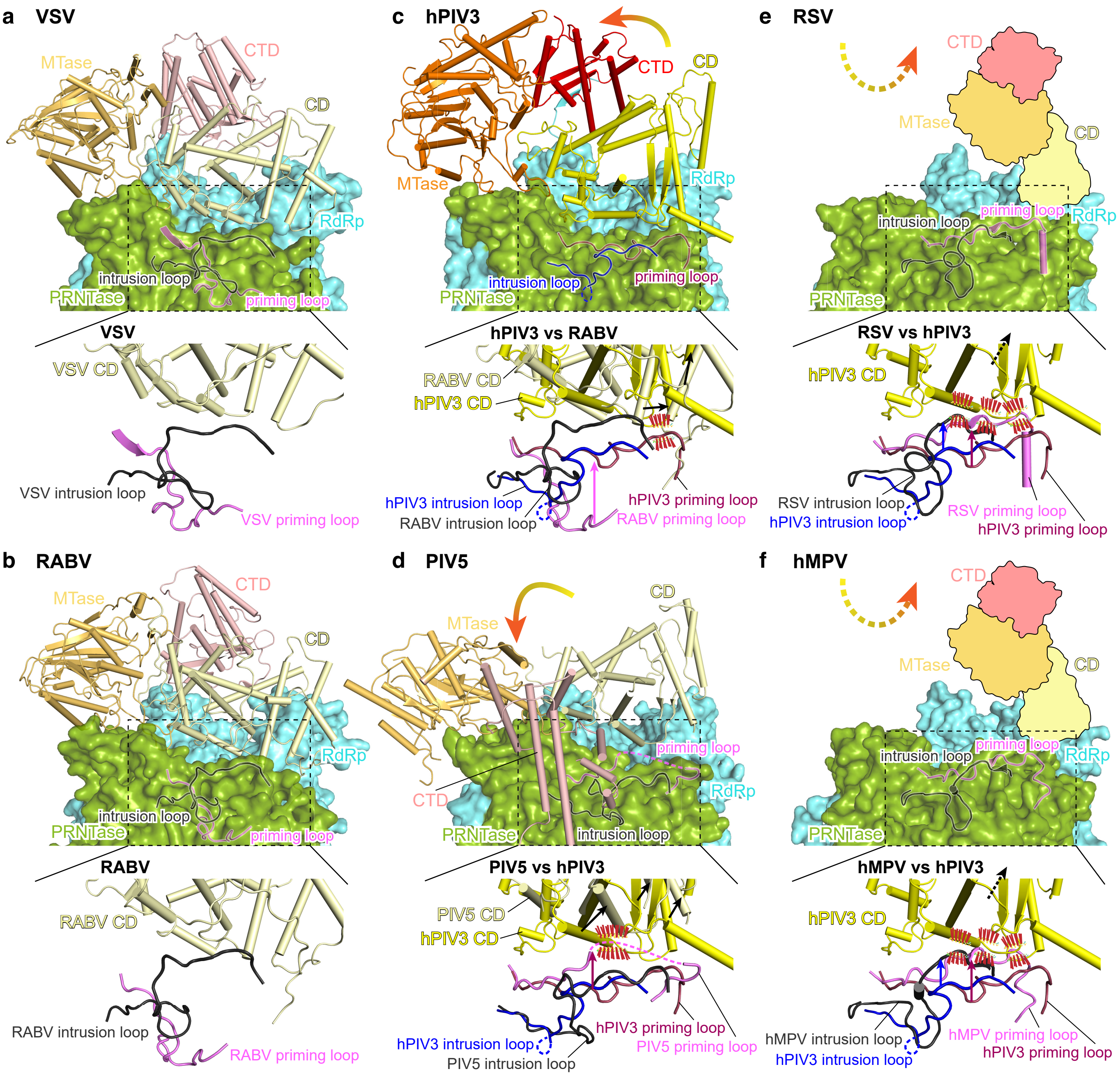

Supplementary Fig. 7. Comparison of L CD-MTase-CTD between different nsNSVs reveals structural correlation between the conformations of priming and intrusion loops and the rearrangements of CD, MTase and CTD domains of L.

a VSV (PDB 6U1X); **b** RABV (PDB 6UEB); **c** hPIV3; **d** PIV5 (PDB 6V85); **e** RSV (PDB 6PZK); **f** hMPV (PDB 6U5O). The putative priming and intrusion loops and the CD domain of hPIV3 L-P structure are compared to those of RABV (**c**), with VSV structure adopting a similar conformation to RABV structure (**a**, **b**). The shifts are indicated by straight arrows, and potential steric clashes between the priming/intrusion loops of hPIV3 structure and the CD domain adopting the same conformation as RABV are indicated by red dashes. The movement of CD-MTase-CTD of hPIV3 compared to that of VSV and RABV in a pre-initiation state[1–3] is indicated by a curved arrow. PIV5 (**d**), RSV (**e**) and hMPV (**f**) L-P structures are compared to that of hPIV3. The CD-MTase-CTD that are invisible in RSV and hMPV L-P structures in a non-initiation state[4–7] are shown as cartoon models with the putative movements shown by dashed arrows.

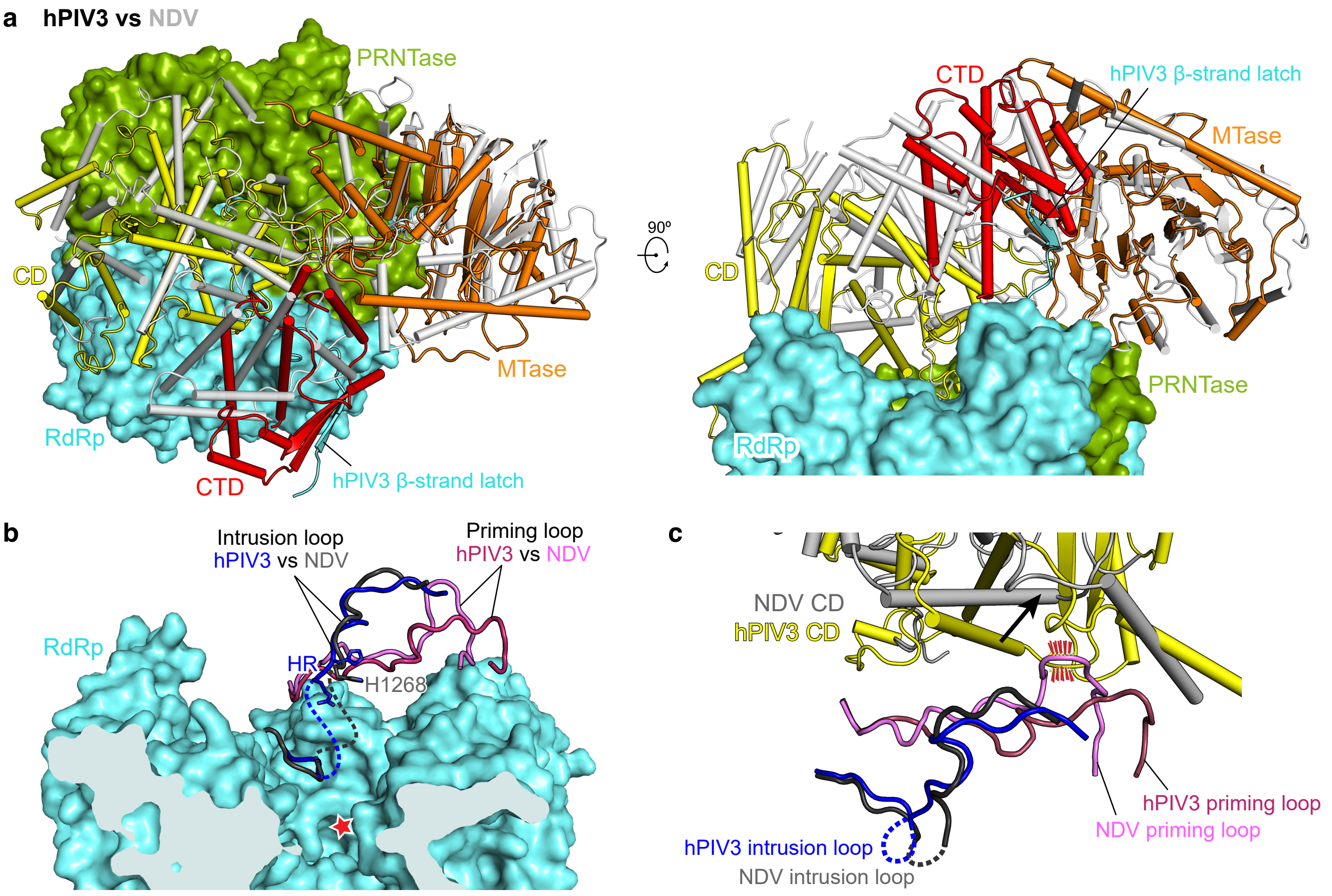

Supplementary Fig. 8. Comparison of hPIV3 L and NDV L structures.

a Comparison of CD-MTase-CTD of hPIV3 (yellow-orange-red) and NDV (PDB 7YOU) (grey). The RdRp-PRNTase domains of hPIV3 and NDV are superimposed and only the hPIV3 RdRp-PRNTase are shown as surface. The β-strand latch of hPIV3 L RdRp is labeled. Rotated view by 90° about the x axis is shown in the right panel. **b** Comparison of the putative priming loops and intrusion loops between hPIV3 and NDV. The disordered regions of the loops are shown as dotted lines. Only His1268 residue of the HR motif of NDV L was built in the EM density map. The hPIV3 RdRp is shown as surface. The red star indicates the RdRp active site. **c** The interactions between the putative priming/intrusion loops and CD. Potential steric clash between NDV priming loop and the CD adopting the same conformation as hPIV3 is indicated by red dashes. The movement is indicated by an arrow.

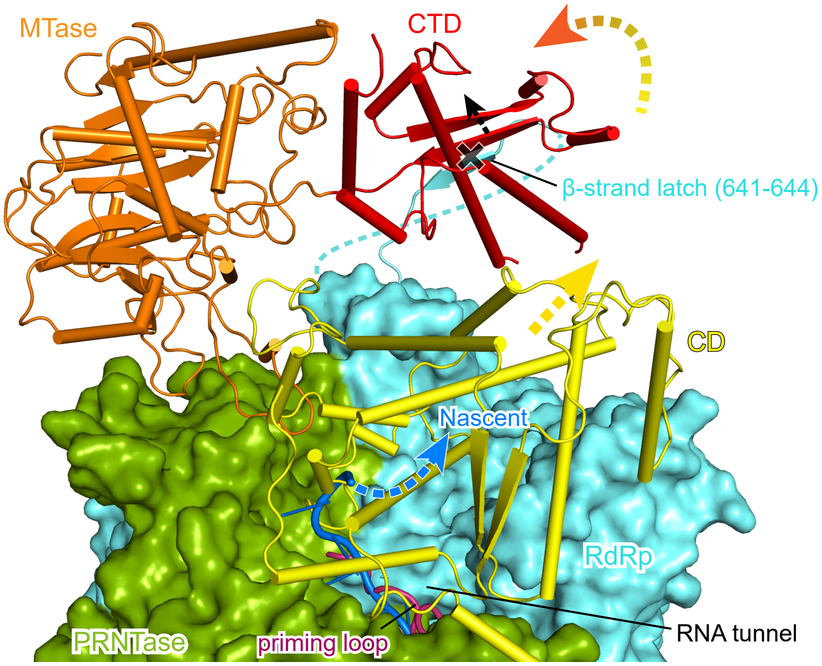

Supplementary Fig. 9. The modeling reveals disruption of the short β-sheet formed with the β-strand latch during elongation.

The domains of hPIV3 L are colored as depicted in Fig. 1a. RdRp and PRNTase domains are shown as surface, while other domains are shown as ribbons. The β-strand latch (Phe641–Lys644) extended from RdRp is labeled, and the disordered region followed by the β-strand latch is shown as a dotted line. The nascent RNA modeled from FluB RdRp structure (PDB 6QCX) is colored in marine blue, and the elongation from the RNA tunnel is indicated by a blue dashed arrow. The part that affects the nascent RNA exit in the priming loop is omitted from the PRNTase surface and shown as ribbons. The shift of the CD domain and the potential movement of CD-MTase-CTD for RNA tunnel opening during elongation are indicated by thick dashed arrows. The disruption of the short β-sheet formed between the β-strand latch and CTD is indicated by a black cross and dashed arrow.

```
hPIV3_P
          1       10        20        30        40         50
hPIV3_P   MESDAKNYQIMDSWEEESRDKSTNISSALNIIEFILSTDPQEDLSEND.TINT......RTQQLS..................
cPIV3_P   MDNNVEDNKIMDSWEERAENRSADISSALDIIEFILNNDTQENANIEN.RIET......GEVDLS..................
hPIV1_P   MDQDAFFFERDPEAEGEAPRKQESLSDVIGLLDVVLSYKPTEIGEDRS.WLHG......IIDNPKENKPS.............
SeV_P     MDQDAFILKEDSEVEREAPGGRESLSDVIGFLDAVLSSEPTDIGGDRS.WLHN......TINTPQGPGS.............A
MeV_P     ................MAEEQARHVKNGLECIRALKAEPIGSLAVEEA.MAAW......SEISDNPGQDR............A
NiV_P     ..................MDKLELVNDGLNIIDFIQKNQKEIQKTYGRSSIQQPSIKDQTKAWEDFLQCTSGESEQVEGGMSK
PIV5_P    ........MDPTDLSFSPDEINKLIETGLNTVEYFTSQQVTGTSSLGKNTIPPGVTGLLTNAAEAKIQE..............
NDV_P     ............MATFTDAEIDELFETSGTVIDSIITAQGKPVETVGRSAIPQGKTKALSLAWEKHGNT..............
RSV_P     ............................MEKFAPEFH..GEDANNRAT.KFLE......SIKGKFT.................
hMPV_P    .........................MSFPEGKDILFM..GNEAAKLAE.AFQK......SLRKPS..................
```

```
hPIV3_P
           60        70        80            90              100                        110
hPIV3_P   .ATIYQPKIKPTETSEKDSGSTDKNRQSG....SSHECT........TEAKDRTID........QETVQR........GPGRR
cPIV3_P   .ATIPYTKSQTTEPYKKDSRSTDKNRWSR....TSHKYT........TKTESGDVD........QEVVQR........RDRRG
hPIV1_P   CKADDNNKDRAISTSTQDHRSSEGSGISR....RTSESK........TETHARILD........QQGIHR........ASRRG
SeV_P     HRAKSEGEGEVSTPSTQDNRSGEESRVSG....RTSKPE........AEAHAGNLD........KQNIHR........AFGGR
MeV_P     TCKEEEAGSSGLSKPCLSAIGSTEGGAPRIRGQGSGESDDD......AET.................................
NiV_P     DDGDVERRNLEDLSSTSPTDGTIGKRVSNTRDWAE.GSDDIQLDPVVTDVVYHDHGGECTGYGFTSSPERGWSDYTSGANNGN
PIV5_P    .STNHQKGSVGGGAKPKKPRPKIAIVPADDK..TV.........P..GKPI................................
NDV_P     .NTPAAQESAGEQDQHGQNQASNSNRATPEEG............PHSSQAQA...............................
RSV_P     .....................SPKD.........PKKKDSIISVNSIDIEVTKE..........SPITSNS..........T.
hMPV_P    ..................................HKRSQSIIGEKVNTVSETLE..........LPTISRP..........TK
```

```
hPIV3_P
               120       130       140       150            160        170        180
hPIV3_P   SSSDSRAETVVSGGISRSITNSKNGTQNTEDIDLNEIRKMDK.....DSIEGKVRQSA.DVPSEISG.SDVIFTTEQSRNSDH
cPIV3_P   SSTDNRIEIMGIGRLPRSIADPEHGSQIQENTDHDEVRKVDK.....DPLEREIRQLE.DVPIKISG.GDTIPSAEQGGDSND
hPIV1_P   TSPNPLPENMGNERNTRIDEDSPNERRHQRSVLTDEDRKMAE.....NSNKREEDQVE.GFPEEVRR.STPLSDDGEGRTNNN
SeV_P     TGTNSVSQDLGDGGDSGILENPPNERGYPRSGIEDENREMAA.....HPDKRGEDQAE.GLPEEVRG.STSLPDEGEGGASNN
MeV_P     .......LGI....PSRNL..QASSTGLQCYHVYDHSGEAVKGIQDADSIMVQS.GLD.G.DSTLSG.GDDESENSDVDIGEP
NiV_P     VCLVSDAKML.............SYAPEIAVSKEDRETDLVHLENK.LSTTGLNPTAVPFTLRNLSDPAKDSPVIAEHYYGLG
PIV5_P    ..........................PNPLLGL.DSTPS............................................
NDV_P     ...................................................................................
RSV_P     ............................IINPTNETDDTAG..........................................
hMPV_P    PTILSEPKLAWTDKGGAIKTEAKQTIK.VMDPIEEEEFT..........EKRVLPSSDGKT.....................P
```

```
hPIV3_P
         190       200                   210        220
hPIV3_P   GRSLESISTPDTRSISVVT.AA...........TPDDEEEI.LMK..N...............................SRTK
cPIV3_P   GGSLEFVSAPYSRGADVIT.IA...........TPSDEEEL.LAK..N...............................TRQR
hPIV1_P   GRSMETSSTHSTRITDVIT.NP...........SPELEDAV.LQR..N...............................KRRP
SeV_P     GRSMEPGSSHSARVTGVLV.IP...........SPELEEAV.LRR..N...............................KRRP
MeV_P     DTEGYAITDRGSAPISMGFRASDVE........TAEGGEIHELLKL.Q.................................SR
NiV_P     VK...........EQNVGPQTSRNVNLDSIKLYTSDDEEADQLEFEDEFAGSSSEVIVGISPEDEEPSSVGGKPNESIGRTIE
PIV5_P    .............TQTVLD.LSG..KTLPSG..SYKGVKLA.KFGKEN...................................
NDV_P     ............ATQPQED.ANE..SQLKTG..ASSSLLSM.LDKLSN...................................
RSV_P     ....NKPNYQRKPLVSFKE.DPT.....PSD.NPFSKLYKETIETFDN...................................
hMPV_P    AEKKLKPSTNTKKKVSFTP.........NEP.GKYTKLEKDALDLLSD...................................
```

```
hPIV3_P
            230              240       250       260                 270
hPIV3_P   KSSSIH..QEDDKRIK.....KGGKGKDWFKKSKDTDNQIPTSDYRS........T..SKGQKK..........ISKTTTI..
cPIV3_P   KNPPEY..QQDNQEIK.....KGKREEKKWSKGERREGQQRREEGEKREREEKIET..GRGIDP..........VPPLAIQ..
hPIV1_P   TTIKRN..QTRSER........................TQSSELHKS........T..SENSSN..........LEDH.NT..
SeV_P     TNS....................................GSKPLTPA........TVPGTRSPP..........LNRYNST..
MeV_P     GNNFPK..LGK...............TLNVPPPPNPSRASTSETPIKKG......TDARLASFG..........TEIASLL..
NiV_P     GQSIRDNLQAKDNKSTDVPGAGPKDSAVKEEPPQKRLPMLAEEFECSGSEDPIIRELLKENSLINCQQGKDAQPPYHWSIERS
PIV5_P    ..........................................................................LMTRFIE..
NDV_P     ..........K...SSN..........AKKGPPQSPPQQA..................................LHSKGSP..
RSV_P     .....N..EEE...SSY...........................................................SYEEI..
hMPV_P    .....N..EEEDAESSI..........................................................LTFEER..
```

```
hPIV3_P
            280       290          300           310       320                       330
hPIV3_P   ....NTDTKGQTEIQTE...SSGTQSSSWNLTI....DNNTDRTEQTNTTPPT................TTSGSTYTKESIRT
cPIV3_P   ....SPQIGASRENQTV...SESQTKRTDPTKT....TGGNQRERPTPLDPCTKGQHTQAHRFSHGAQRSTGDQSGTFRPKVS
hPIV1_P   ....KTSPKVPPSKNEE...SAATPKNNHNHRK....TRYT..................................TNNANNNT
SeV_P     ....GSPPGKPPSTQDEHINSGDTPAVRVKDRKPPIGTRSV..................................SDCPANGR
MeV_P     ......................................................................TGGATQCARKSPS
NiV_P     ISPDKTEIVNGAVQTADRQRPGTPMPKSRGIPIKK.GTDAKYPSAGTENVPG.................SKSGATRHVRGSPP
PIV5_P    ....EPRENPIATS.......SPIDFKRGAGIPAG.SIEGSTQSDGWEMKSR.................SLSGAIHPV.....
NDV_P     ....AVEQTQHGANQGR..AQQETGHQAAPSPGPP.GTGVNIAFPGQRGVSP.................QSVGATQPA.....
RSV_P     ....NDQTNDN........................................................................
hMPV_P    ....DTSSL..........................................................................
```

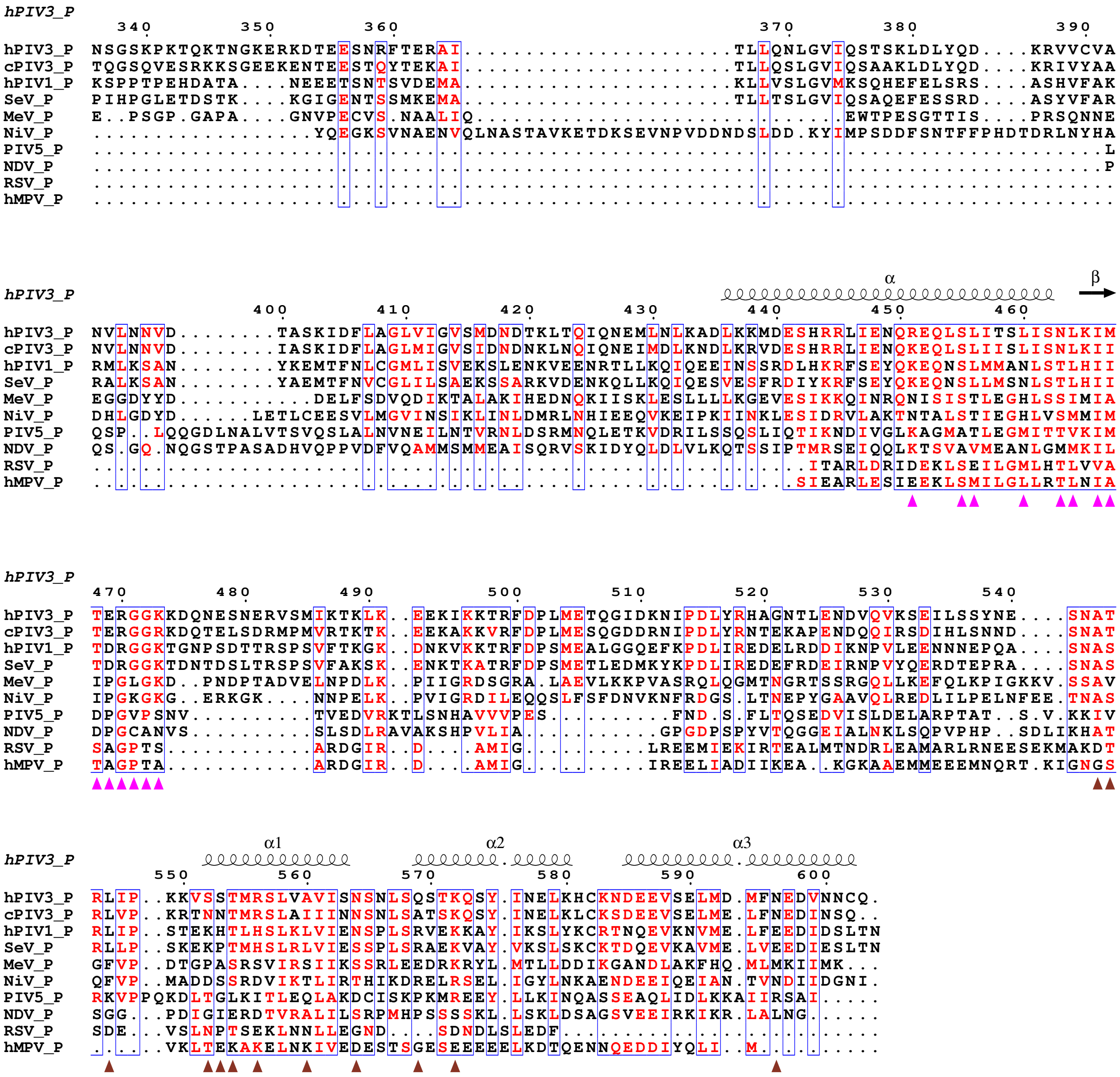

Supplementary Fig. 10. Structure-based sequence alignment of nsNSV P proteins.

Secondary structure elements of hPIV3 P (P4-OD and single XD) are shown above the alignment. The residues of hPIV3 P-OD and P-XD involved in the interactions with L are indicated by pink and maroon triangles, respectively. The sequences of P proteins are from the following viruses: hPIV3 (NP_067149.1), cPIV3 (YP_009179208.1), hPIV1 (NP_604435.1), SeV (BAN84668.1), MeV (NP_056919.1), NiV (NP_112022.1), PIV5 (YP_138512.1), NDV (ALP75892.1), RSV (YP_009518853.1) and hMPV (Q8B9Q8).

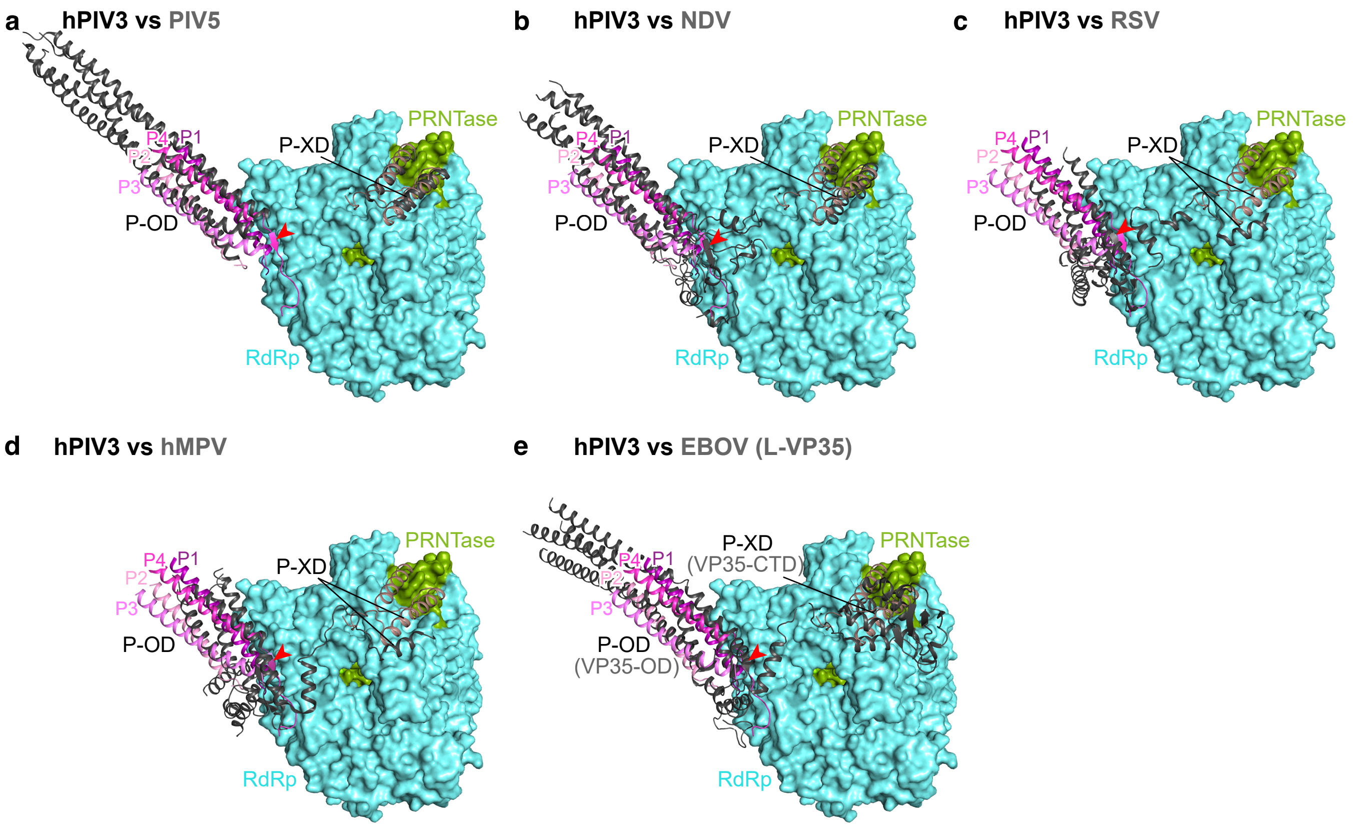

Supplementary Fig. 11. Comparison of L-P binding between hPIV3 and other nsNSVs including PIV5 (a), NDV (b), RSV (c), hMPV (d) and EBOV (L-VP35) (e).

The RdRp and PRNTase structures of PIV5 (PDB 6V85), NDV (PDB 7YOV), RSV (PDB 6PZK), hMPV (PDB 6U5O) and EBOV (PDB 7YES) are superimposed to that of hPIV3 with surfaces of hPIV3 RdRp and PRNTase shown. HPIV3 L and P are colored as depicted in Fig. 4a, while P or VP35 proteins of other nsNSVs are colored in dark grey. Both OD and XD domains of hPIV3, PIV5 and NDV P proteins as well as the OD and CTD domains of EBOV VP35 adopt similar conformations and bind to the same approximate positions on L (**a**, **b** and **e**). RSV and hMPV also dock the tetrameric helical OD domains of P on the L surface like hPIV3 and extend their C-terminal helices of one monomer to roughly the same position as hPIV3 P-XD (**c**, **d**). Red arrows indicate the typical antiparallel β-sheet between L and P-OD shared among hPIV3, NDV, RSV, hMPV and EBOV (between L and VP35-OD), while it was not resolved in PIV5 structure due to low resolution.

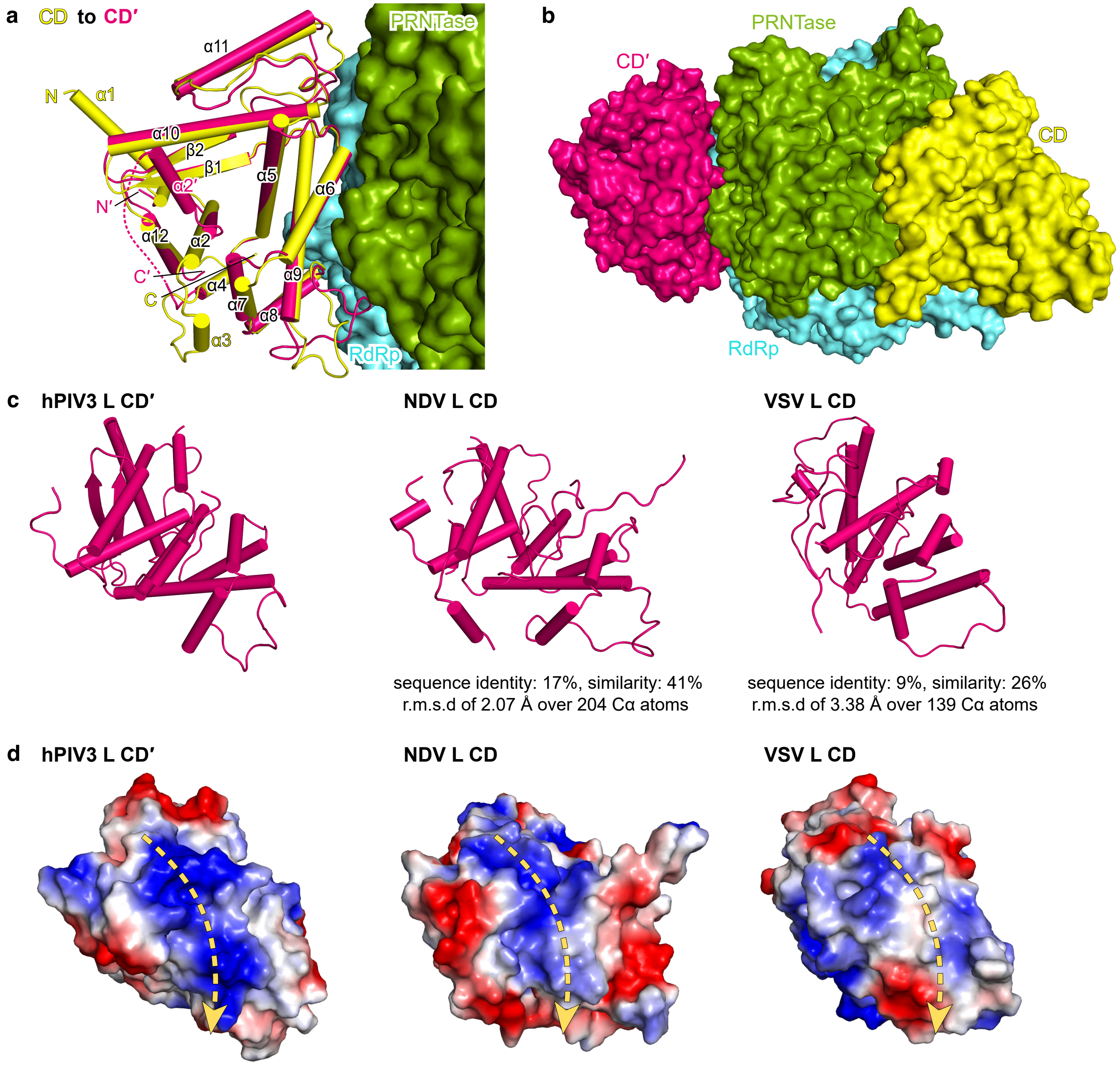

Supplementary Fig. 12. The CD domain (CD′) of the second L of hPIV3.

a CD is superimposed to CD′ with the secondary structure elements and both N and C termini labeled, and RdRp and PRNTase domains shown as surface. The first α-helix (α1) is invisible in CD′. The disordered region containing the missing short helix α3 of CD′ is shown as a dotted line. The major structural differences are the loops between α8/α9 and α11/β2 at the L-L interface and the bent helix α2 of CD′ (α2′). **b** Two CD domains located at both sides of RdRp-PRNTase of L. All domains are shown as surface. **c** Structural similarity between hPIV3 L CD′ (left) and the CD domains of another paramyxovirus NDV L (PDB 7YOU) and the rhabdovirus VSV L (PDB 6U1X). Structures were superimposed to CD′ of hPIV3 L, and the r.m.s.d value and residue coverage are marked. The sequence identities and similarities of the CD domains compared to that of hPIV3 are also shown. **d** The positively charged surface on CD′ of hPIV3 L (left) and the comparable surface on the CD domains of NDV L and VSV L at the same top view as in Fig. 7f. The putative RNA paths are indicated by dashed arrows.

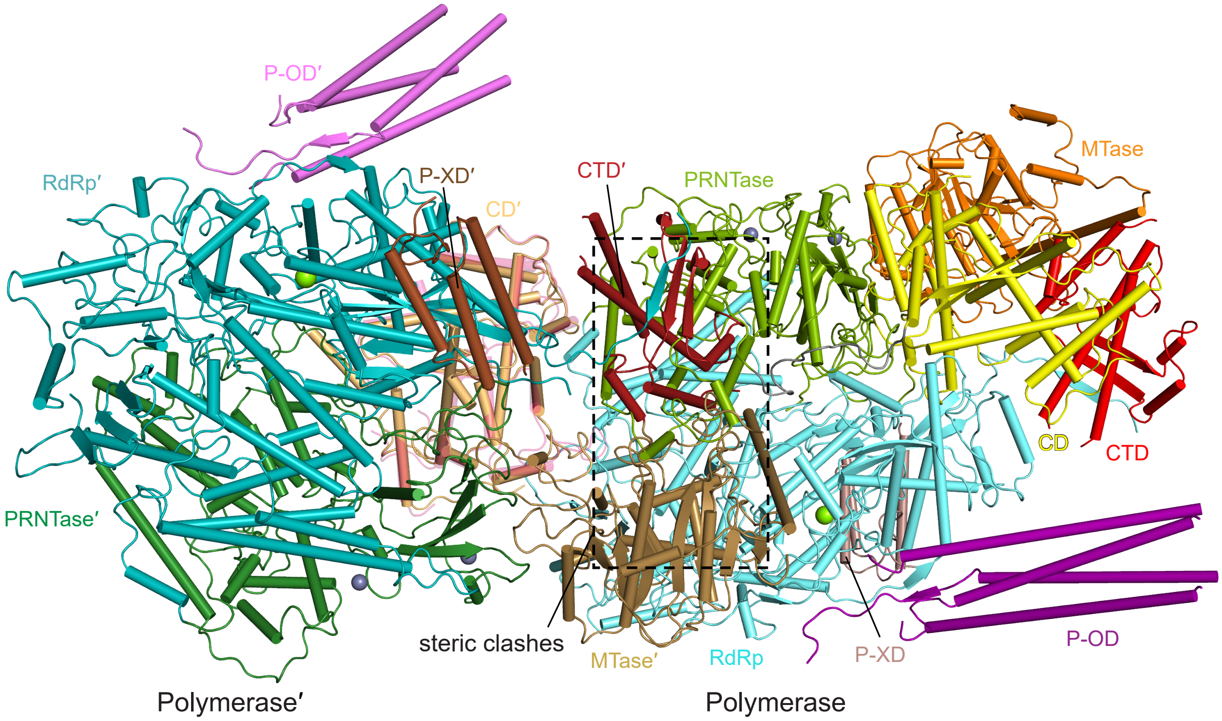

Supplementary Fig. 13. The captured hPIV3 L-P dimer should not be symmetric.

Dimer modeling using two same hPIV3 L-P monomers by superposition of the CD domain of the second L-P to the second CD (CD′) in the incomplete dimeric hPIV3 L-P structure showed that two L-P monomers have serious steric clashes, shown in the dashed box. The original L-P of Polymerase is colored as depicted in Fig. 1a, while the superimposed L-P of Polymerase′ is drawn in close colors.

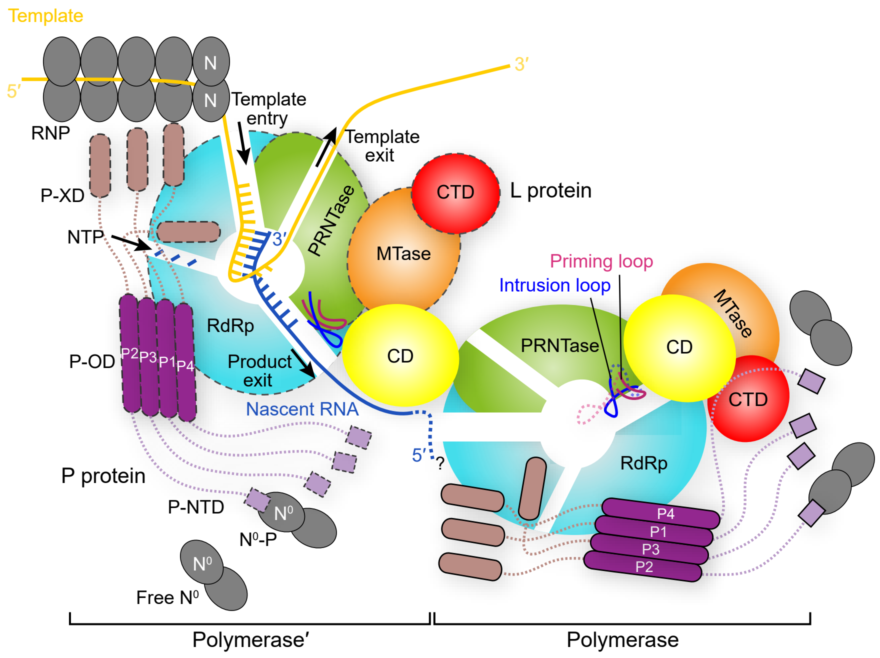

Supplementary Fig. 14. The dimeric L-P polymerase model in RNA replication.

Schematic diagram illustrating a speculative model for the function of the dimeric L-P polymerase. In this schematic diagram, the right-hand polymerase represents the L-P complex for which an almost intact structure was obtained by cryo-EM, and the left-hand polymerase represents the L-P complex for which most parts were unresolved, except for the CD domain. It should be noted that the cartoon is not a precise 2D projection but is a "flattened" representation so that the RNA and NTP channels of both polymerases could be depicted. According to this model, the left-hand polymerase (Polymerase′) contacts the template and synthesizes RNA. The retracted priming loop into the cavity of the PRNTase, together with the intrusion loop, pushes away the CD with dramatic movement of the adjacent C-terminal MTase-CTD, resulting in a dynamic structure. The opening of the product exit channel allows nascent RNA to travel along the positively charged surface of CD′ towards the right-hand polymerase. The right-hand polymerase might play a role in encapsidating the RNA in the presence of soluble N^0, although the site of encapsidation is not known (as indicated with a dotted line and a question mark). In this model, the unresolved parts of the left-hand polymerase in the dimeric hPIV3 L-P cryo-EM structure are highlighted by dashed strokes. The putative priming and intrusion loops in pre-initiation state are indicated by dashed transparent lines. For simplicity, soluble N^0 is shown in the free state, but in infected cells, it would likely be in an N^0-P complex.

Supplementary Table 1. Cryo-EM data collection, structure refinement and model statistics

	hPIV3 L:P complex class 1 (incomplete dimeric form)	hPIV3 L:P complex class 2 (monomeric form)
**PDB entry**	8KDB	8KDC
**EMDB entry**	EMD-37130	EMD-37131
**Data collection and processing**		
Magnification	81,000	81,000
Voltage (kV)	300	300
Electron exposure (e^{-}/Å^2)	50	50
Defocus range (μm)	−1.5 to −2.0	−1.5 to −2.0
Pixel size (Å)	1.087	1.087
Symmetry imposed	C1	C1
Initial particles (no.)	3,288,374	3,288,374
Final particles (no.)	102,956	137,294
Map resolution (Å)	2.7	3.3
FSC threshold	0.143	0.143
Map resolution range (Å)	∞–2.7	∞–3.3
**Refinement**		
Initial model used (PDB code)	6V85	6V85
Map sharpening *B* factor (Å^2)	-45	-109
Model composition		
Non-hydrogen atoms	20,861	18,808
Protein residues	2,584	2,329
Ligands	Mg^{2+} (1), Zn^{2+} (2)	Mg^{2+} (1), Zn^{2+} (2)
B factors (Å^2)		
Protein	70.77	92.68
Ligand	70.00	104.78
R.m.s. deviations		
Bond lengths (Å)	0.003	0.003
Bond angles (°)	0.525	0.495
Validation		
MolProbity score	1.28	1.48
Clashscore	5.27	6.45
Poor rotamers (%)	0.04	0.05
Ramachandran plot		
Favored (%)	98.01	97.36
Allowed (%)	1.99	2.64
Disallowed (%)	0.00	0.00

Supplementary references

1. Liang, B. *et al.* Structure of the L protein of vesicular stomatitis virus from electron cryomicroscopy. *Cell* **162**, 314–327 (2015).

2. Jenni, S. *et al.* Structure of the vesicular stomatitis virus L protein in complex with its phosphoprotein cofactor. *Cell Rep.* **30**, 53-60.e5 (2020).

3. Horwitz, J. A., Jenni, S., Harrison, S. C. & Whelan, S. P. J. Structure of a rabies virus polymerase complex from electron cryo-microscopy. *Proc. Natl. Acad. Sci. U. S. A.* **117**, 2099–2107 (2020).

4. Pan, J. *et al.* Structure of the human metapneumovirus polymerase phosphoprotein complex. *Nature* **577**, 275–279 (2020).

5. Gilman, M. S. A. *et al.* Structure of the respiratory syncytial virus polymerase complex. *Cell* **179**, 193-204.e14 (2019).

6. Te Velthuis, A. J. W., Grimes, J. M. & Fodor, E. Structural insights into RNA polymerases of negative-sense RNA viruses. *Nat. Rev. Microbiol.* **19**, 303–318 (2021).

7. Cao, D. *et al.* Cryo-EM structure of the respiratory syncytial virus RNA polymerase. *Nat. Commun.* **11**, 1–9 (2020).
