# Supplementary material for: Structural basis for dimerization of a paramyxovirus polymerase complex
Source: Nat Commun. 2024 Apr 11;15:3163. doi: 10.1038/s41467-024-47470-7 (PMC11009304; doi:10.1038/s41467-024-47470-7)
Supplement: Supplementary file 3 — Reporting Summary [file 41467_2024_47470_MOESM3_ESM.pdf]

nature portfolio

Corresponding author(s): Lu Gao, Xinyi Huang, Rachel Fearns, Shuai Chen

Last updated by author(s): Mar 11, 2024

Reporting Summary

Nature Portfolio wishes to improve the reproducibility of the work that we publish. This form provides structure for consistency and transparency in reporting. For further information on Nature Portfolio policies, see our Editorial Policies and the Editorial Policy Checklist.

Statistics

For all statistical analyses, confirm that the following items are present in the figure legend, table legend, main text, or Methods section.

n/a	Confirmed	
☐	☒	The exact sample size (*n*) for each experimental group/condition, given as a discrete number and unit of measurement
☐	☒	A statement on whether measurements were taken from distinct samples or whether the same sample was measured repeatedly
☒	☐	The statistical test(s) used AND whether they are one- or two-sided<br>*Only common tests should be described solely by name; describe more complex techniques in the Methods section.*
☒	☐	A description of all covariates tested
☒	☐	A description of any assumptions or corrections, such as tests of normality and adjustment for multiple comparisons
☐	☒	A full description of the statistical parameters including central tendency (e.g. means) or other basic estimates (e.g. regression coefficient) AND variation (e.g. standard deviation) or associated estimates of uncertainty (e.g. confidence intervals)
☒	☐	For null hypothesis testing, the test statistic (e.g. *F*, *t*, *r*) with confidence intervals, effect sizes, degrees of freedom and *P* value noted<br>*Give P values as exact values whenever suitable.*
☒	☐	For Bayesian analysis, information on the choice of priors and Markov chain Monte Carlo settings
☒	☐	For hierarchical and complex designs, identification of the appropriate level for tests and full reporting of outcomes
☒	☐	Estimates of effect sizes (e.g. Cohen's *d*, Pearson's *r*), indicating how they were calculated

Our web collection on statistics for biologists contains articles on many of the points above.

Software and code

Policy information about availability of computer code

Data collection	AutoEMation
Data analysis	RELION 3.1, cryoSPARC 3.1.0, Coot 0.9, PHENIX (version dev-4230), UCSF Chimera 1.13.1 and ChimeraX, PyMOL 1.8, MolProbity 4.5, Multalin 5.4.1, PROMALS3D, GraphPad Prism 8, ASTRA 6.1

For manuscripts utilizing custom algorithms or software that are central to the research but not yet described in published literature, software must be made available to editors and reviewers. We strongly encourage code deposition in a community repository (e.g. GitHub). See the Nature Portfolio guidelines for submitting code & software for further information.

Data

Policy information about availability of data

All manuscripts must include a data availability statement. This statement should provide the following information, where applicable:

- Accession codes, unique identifiers, or web links for publicly available datasets
- A description of any restrictions on data availability
- For clinical datasets or third party data, please ensure that the statement adheres to our policy

All data supporting the findings of this study are available in the manuscript and the supplementary materials. Source data are provided with this paper. The cryo-EM density maps generated in this study have been deposited in the Electron Microscopy Data Bank (EMDB) under accession codes EMD-37130 [https://www.ebi.ac.uk/emdb/EMD-37130] (hPIV3 L-P class 1, incomplete dimeric form) and EMD-37131 [https://www.ebi.ac.uk/emdb/EMD-37131] (hPIV3 L-P class 2, monomeric form). The corresponding atomic coordinates have been deposited in the Protein Data Bank (PDB) under accession codes 8KDB [https://

doi.org/10.2210/pdb8kdb/pdb] (hPIV3 L-P class 1, incomplete dimeric form) and 8KDC [https://doi.org/10.2210/pdb8kdc/pdb] (hPIV3 L-P class 2, monomeric form). The previously published structures used for comparison and analysis in this study are available in the PDB under the following accession codes: 6PZK [https://doi.org/10.2210/pdb6pzk/pdb], 6QCX [https://doi.org/10.2210/pdb6qcx/pdb], 6U1X [https://doi.org/10.2210/pdb6u1x/pdb], 6U5O [https://doi.org/10.2210/pdb6u5o/pdb], 6UEB [https://doi.org/10.2210/pdb6ueb/pdb], 6V85 [https://doi.org/10.2210/pdb6v85/pdb], 7BV2 [https://doi.org/10.2210/pdb7bv2/pdb], 7YES [https://doi.org/10.2210/pdb7yes/pdb], 7YOU [https://doi.org/10.2210/pdb7you/pdb], 7YOV [https://doi.org/10.2210/pdb7yov/pdb], 8JSN [https://doi.org/10.2210/pdb8jsn/pdb] and 8SNX [https://doi.org/10.2210/pdb8snx/pdb].

Research involving human participants, their data, or biological material

Policy information about studies with human participants or human data. See also policy information about sex, gender (identity/presentation), and sexual orientation and race, ethnicity and racism.

Reporting on sex and gender	N/A
Reporting on race, ethnicity, or other socially relevant groupings	N/A
Population characteristics	N/A
Recruitment	N/A
Ethics oversight	N/A

Note that full information on the approval of the study protocol must also be provided in the manuscript.

Field-specific reporting

Please select the one below that is the best fit for your research. If you are not sure, read the appropriate sections before making your selection.

- [x] Life sciences
- [ ] Behavioural & social sciences
- [ ] Ecological, evolutionary & environmental sciences

For a reference copy of the document with all sections, see nature.com/documents/nr-reporting-summary-flat.pdf

Life sciences study design

All studies must disclose on these points even when the disclosure is negative.

Sample size	A total number of 5,800 cryo-EM movie stacks were collected. 5,693 micrographs were selected for subsequent processing, and 102,956 and 137,294 particles were extracted for reconstruction of the high-resolution cryo-EM maps of hPIV3 L-P class 1 (incomplete dimeric form, 2.7 Å) and class 2 (monomeric form, 3.3 Å), respectively. The functional data were generated from two or three independent experiments as described in relevant figure legends.
Data exclusions	Following the first steps of cryo-EM data processing, some cryo-EM micrographs with poor quality were discarded.
Replication	All functional assays were carried out at least two times and produced similar results.
Randomization	For cryo-EM data processing, the datasets were randomly split into two halves for unbiased 3D reconstruction. The biochemical and cellular assays in this study do not involve randomization, as they do not contain experiments with groups of individuals.
Blinding	Blinding is unnecessary and not applicable for cryo-EM structure determination and in vitro assays in this study.

Reporting for specific materials, systems and methods

We require information from authors about some types of materials, experimental systems and methods used in many studies. Here, indicate whether each material, system or method listed is relevant to your study. If you are not sure if a list item applies to your research, read the appropriate section before selecting a response.

Materials & experimental systems

n/a	Involved in the study	
☐	☒	Antibodies
☐	☒	Eukaryotic cell lines
☒	☐	Palaeontology and archaeology
☒	☐	Animals and other organisms
☒	☐	Clinical data
☒	☐	Dual use research of concern
☒	☐	Plants

Methods

n/a	Involved in the study	
☒	☐	ChIP-seq
☒	☐	Flow cytometry
☒	☐	MRI-based neuroimaging

Antibodies

Antibodies used	Rabbit anti-FLAG monoclonal antibody (Cell Signaling Technology, 14793S, clone D6W5B, 1:1,000 dilution), mouse anti-GAPDH monoclonal antibody (Proteintech, 60004-1-Ig, clone 1E6D9, 1:2,000 dilution), donkey anti-rabbit IRDye 680RD antibody (LI-COR, 926-68073, 1:20,000 dilution) and goat anti-mouse IRDye 800CW antibody (LI-COR, 926-32210, 1:20,000 dilution).
Validation	All antibodies used in this study were validated by the manufacturer. The anti-GAPDH antibody has also been validated by KO/KD. In our experiments we included a negative control of untagged L protein to confirm the specificity of the anti-FLAG antibody (anti-DYKDDDDK Tag) under our experimental conditions.<br>Rabbit anti-FLAG monoclonal antibody https://www.cellsignal.cn/products/primary-antibodies/dykddddk-tag-d6w5b-rabbit-mab-binds-to-same-epitope-as-sigma-s-anti-flag-m2-antibody/14793<br>Mouse anti-GAPDH monoclonal antibody https://www.ptglab.com/products/GAPDH-Antibody-60004-1-Ig.htm<br>Donkey anti-rabbit IRDye 680RD antibody https://www.licor.com/bio/reagents/irdye-680rd-donkey-anti-rabbit-igg-secondary-antibody<br>Goat anti-mouse IRDye 800CW antibody https://www.licor.com/bio/reagents/irdye-800cw-goat-anti-mouse-igg-secondary-antibody

Eukaryotic cell lines

Policy information about cell lines and Sex and Gender in Research

Cell line source(s)	The human laryngeal cancer cell line (HEp-2) was purchased from ATCC (CCL-23). The Sf21 insect cell line was purchased from Thermo Fisher Scientific.
Authentication	The cells were purchased and routinely maintained. They were not authenticated experimentally for this study.
Mycoplasma contamination	Mycoplasma testing revealed no contamination.
Commonly misidentified lines<br>(See ICLAC register)	HEp-2 cell line is a HeLa cell contaminant. HEp-2 cells are used routinely in the Fearns lab as a host for minigenome assays to study viral transcription and replication using the protocol followed in the present work. These cells are used because they are readily transfectable and support efficient transcription and replication by the viral polymerase, the processes that are under investigation in this study. Thus, the contaminating HeLa cells are not expected to influence the results reported here.

Plants

Seed stocks	N/A
Novel plant genotypes	N/A
Authentication	N/A
